# Transcriptomic analysis of Siberian ginseng (*Eleutherococcus senticosus*) to discover genes involved in saponin biosynthesis

**DOI:** 10.1186/s12864-015-1357-z

**Published:** 2015-03-14

**Authors:** Hwan-Su Hwang, Hyoshin Lee, Yong Eui Choi

**Affiliations:** Department of Forest Resources, College of Forest and Environmental Sciences, Kangwon National University, Chunchun, 200-701 South Korea; Biotechnology Division, Korea Forest Research Institute, Suwon, 441-350 South Korea

**Keywords:** Cytochrome P450, UDP-glycosyltransferase, Saponin, Transcriptome analysis, De novo sequencing, Next-generation sequencing, *Eleutherococcus senticosus*

## Abstract

**Background:**

*Eleutherococcus senticosus,* Siberian ginseng, is a highly valued woody medicinal plant belonging to the family Araliaceae. *E. senticosus* produces a rich variety of saponins such as oleanane-type, noroleanane-type, 29-hydroxyoleanan-type, and lupane-type saponins. Genomic or transcriptomic approaches have not been used to investigate the saponin biosynthetic pathway in this plant.

**Result:**

In this study, *de novo* sequencing was performed to select candidate genes involved in the saponin biosynthetic pathway. A half-plate 454 pyrosequencing run produced 627,923 high-quality reads with an average sequence length of 422 bases. *De novo* assembly generated 72,811 unique sequences, including 15,217 contigs and 57,594 singletons. Approximately 48,300 (66.3%) unique sequences were annotated using BLAST similarity searches. All of the mevalonate pathway genes for saponin biosynthesis starting from acetyl-CoA were isolated. Moreover, 206 reads of cytochrome P450 (CYP) and 145 reads of uridine diphosphate glycosyltransferase (UGT) sequences were isolated. Based on methyl jasmonate (MeJA) treatment and real-time PCR (qPCR) analysis, 3 CYPs and 3 UGTs were finally selected as candidate genes involved in the saponin biosynthetic pathway.

**Conclusions:**

The identified sequences associated with saponin biosynthesis will facilitate the study of the functional genomics of saponin biosynthesis and genetic engineering of *E. senticosus*.

**Electronic supplementary material:**

The online version of this article (doi:10.1186/s12864-015-1357-z) contains supplementary material, which is available to authorized users.

## Background

*Eleutherococcus senticosus* Maxim (= *Acanthopanax senticosus*) is a thorny shrub belonging to Araliaceae that grows in the Russian Far East, Northeast China, Korea and Japan. There are approximately 38 species of *Eleutherococcus. E. senticosus* is popularly known as Siberian ginseng because of its remarkable pharmacological effects. The cortical root and stem tissues of the plant are used as a tonic and sedative and to treat rheumatism and diabetes [1,2]. Its main ingredients are triterpenoid saponins, lignans, and phenolic compounds [3].

*E. senticosus* produces various types of triterpene saponins. Huang et al. [3] reviewed 43 types of triterpene saponins isolated from *E. senticosus*. The representative saponins of *E. senticosus* are oleanane-type triterpene saponins (referred to as eleutherosides I, K, L, and M and ciwujianoside A1, C3, C4, and D1). Moreover, noroleanane-type (ciwujianoside A2, B, C1, C2, D2, and E), 29-hydroxyoleanan type (ciwujianoside A3, A4, and D3) and lupane-type triterpene saponins (chiisanoside) have been isolated from *E. senticosus* [4].

Saponin synthesis starts from the acetylated coenzyme A (acetyl CoA) molecule, from which all triterpene carbon atoms are derived. The first diversifying step in triterpenoid biosynthesis is the cyclisation of 2,3-oxidosqualene catalysed by oxidosqualene cyclase (OSC) [5]. The molecular diversity of OSCs enables more than 100 skeletal variations of triterpenoids in plants [6]. Saponins are thought to be synthesised from subsequent hydroxylation or oxidation of triterpene skeletons by CYP and glycosylation by UGT. These enzymes exist as supergene families in the plant genome. However, the key genes involved in saponin biosynthesis in *E. senticosus* have not been identified.

Expressed sequence tag (EST) analysis is a powerful method to discover novel genes [7]. Next-generation sequencing (NGS) technologies have enabled a genomics and genetics revolution in which the discovery of useful genes has been greatly accelerated [8,9]. NGS sequencing has been used in saponin-rich plant species such as the *Panax* species [10,11], *Siraitia grosvenorii* [12], and *Buplerum chinense* [13], and *Ilex asperlla* [14] to identify triterpene biosynthetic genes.

Despite the economic and pharmacological value of *E. senticosus*, it has not been characterised using genomic and transcriptomic approaches. In this research, 627,923 reads were generated using the Roche GS FLX titanium platform from a leaf cDNA library from *E. senticosus*. The reads were assembled to 15,217 contigs and 57,594 singletons. We focused on discovering genes encoding enzymes involved in the saponin biosynthesis pathway. Genes involved in saponin skeleton biosynthesis as well as a number of candidate genes that might be involved in modification of the triterpene saponin biosynthetic pathway skeleton, including CYPs and UGTs, were screened by elicitor treatment. Candidate CYP and UGT genes were selected based on their putative involvement in saponin biosynthesis in *E. senticosus*.

## Results

### Sequencing using the 454 genome sequencer FLX system and *de novo* assembly

**Figure 1:**
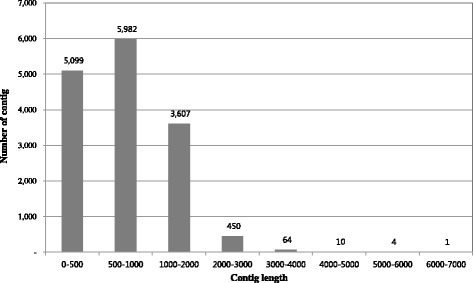
**Length distribution of the assembled contigs of** ***E. senticosus*** **.**

A cDNA library constructed from total RNA extracted from *E. senticosus* leaves was sequenced on a one-half plate using the GS FLX Titanium platform. After trimming adapter sequences and removing repeat sequences or short sequences of less than 50 bp, a total of 627,923 reads were generated as 371,784 reads with an average length of 422 bp. The 371,784 reads were then used for assembly by Roche Newbler Software as 15,217 contigs and 57,594 singletons. The longest contig was 6,537 bp, with an average total contig length of 785 bp. The singletons ranged in size from 50 to 948 bp, with an average length of 368 bp. Information on bases, contigs and singletons is presented in Table 1. The size distribution of the contigs is shown in Figure 1.

**Table 1:** **Summary of the total 454 sequencing and the assembly results for** ***E. senticosus*** **leaf tissues**

**Items**	**No. of sequences**	**No. of bases**
Total number of reads	627,923	264,936,636
Average read length (bp)	422	
Reads used in assembly	375,482 (59.80%)	145,595,016 (54.95%)
Number of contigs	15,217	11,958,995
Average length of contigs (bp)	785	
Range of contig length (bp)	100-6,537	
Number of singletons (bp)	57,594	21,194,393
Average length of singletons (bp)	368	
Range of singleton length (bp)	50-948

### Functional annotation and classification based on gene ontology (GO)

The unique sequences were compared with the NCBI non-redundant nucleotide database (Nt) and three major protein databases (KEGG, Nr, and UniProt) using the BLASTN and BLASTX algorithms with an E-value cutoff of < 10^−5^. A total of 48,300 (66.3%) unique sequences with a significant match were annotated (Table 2).

**Table 2:** **Summary of the annotation of the 454 assembled unique sequences**

**Annotation database**	**Annotation number**	**Annotation percentage (%)**
KEGG	43,041	59.1
Nt	40,712	55.9
Nr	44,712	61.4
UniProt	43,300	59.5
Total	48,300	66.3

The annotations were obtained by comparing the assembled sequences with sequences from KEGG, Nr, and UniProt of public databases.

**Figure 2:**
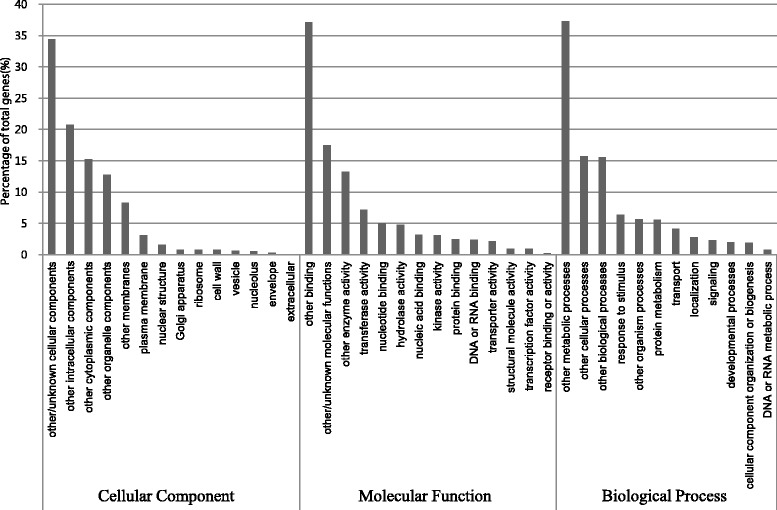
**Histogram presentation of functional annotations of the unique sequences by the gene ontology classification.** The results are summarised in three main categories: cellular component, molecular function and biological process. The right y-axis indicates the number of genes in a category. The left y-axis indicates the percentage of unique sequences in a specific category.

The nineteen sequences listed in Table 3 are the most abundant transcripts in the 454 cDNA library, with greater than 2,000 reads. These include the genes encoding ATP synthase, chlorophyll a/b binding protein, cell wall-associated hydrolase, and cytochrome P450. The most abundant transcript, with 7,284 reads, was annotated as a chloroplast-unknown-protein. Gene ontology (GO) analysis revealed three major categories: biological process, molecular function and cellular component. A total of 41,746 (53.4%) of unique sequences were annotated based on The Arabidopsis Information Resource (TAIR) proteins and assigned using gene ontology terms (Figure 2). The major groups of the molecular function category were transferase activity, nucleotide binding, hydrolase activity, nucleic acid binding, and kinase activity. In the cellular component group, many sequences were annotated as plasma membrane, nuclear structure, and Golgi apparatus. The best represented groups were response to the stimulus, protein metabolism, and transport in biological process categories.

**Table 3:** **Most abundant transcripts in** ***E. senticosus*** **leaf transcriptome**

**Contig ID**	**Length (bp)**	**Read**	**Target accession no.**	**Target description**
EPT001TT0600C012714	253	7284	KC844054	Chloroplast, complete genome [*Aconitum barbatum*]
EPT001TT0600C012563	263	7080	KF856619	Chloroplast, complete genome [*Cercis canadensis*]
EPT001TT0600C015018	111	5674	CAA42617	Ribulose bisphosphate carboxylase [*Phaseolus vulgaris*]
EPT001TT0600C012209	292	4515	CAN59721	Hypothetical protein VITISV_032350 [*Vitis vinifera*]
EPT001TT0600C000003	5621	3273	XP_003621695	ATP synthase subunit beta [*Medicago truncatula*]
EPT001TT0600C013281	215	3222	ZP_06388631	Hypothetical protein Ssol98_08391 [*Sulfolobus solfataricus*]
EPT001TT0600C012224	291	3026	AFO67221	Putative chlorophyll a/b binding protein, partial [*Aralia elata*]
EPT001TT0600C012058	305	2893	BAE46384	Ribulose-1,5-bisphosphate carboxylase/oxygenase small subunit [*Panax ginseng*]
EPT001TT0600C014082	163	2852	XP_003637074	Cell wall-associated hydrolase, partial [*Medicago truncatula*]
EPT001TT0600C014525	137	2717	XP_003544026	PREDICTED: uncharacterised protein LOC100801029 [*Glycine max*]
EPT001TT0600C008793	616	2650	CAA48410	Light harvesting chlorophyll a /b binding protein [*Hedera helix*]
EPT001TT0600C013420	206	2624	BAE46384	Ribulose-1,5-bisphosphate carboxylase/oxygenase small subunit [*Panax ginseng*]
EPT001TT0600C013790	181	2602	BAD26579	Cytochrome P450-like TBP [*Citrullus lanatus*]
EPT001TT0600C011532	359	2376	AFO67218	Putative ribulose-1,5-bisphosphate carboxylase/oxygenase small subunit [*Aralia elata*]
EPT001TT0600C014447	141	2347	XP_003064992	Senescence-associated protein [*Micromonas pusilla*]
EPT001TT0600C010797	426	2319		No hit
EPT001TT0600C001408	1540	2219	XP_003588355	Mitochondrial protein, putative [*Medicago truncatula*]
EPT001TT0600C011162	393	2219	XP_003637074	Cell wall-associated hydrolase, partial [*Medicago truncatula*]
EPT001TT0600C015121	106	2045	ZP_04821298	Conserved hypothetical protein [*Clostridium botulinum*]

### Mevalonate pathway genes as candidates for involvement in saponin backbone biosynthesis

Triterpenes are assembled from a five-carbon isoprene unit through the cytosolic mevalonate pathway. Mevalonate is a product of the sequential condensation of three acetyl-CoA units to generate 3-hydroxy-3-methylglutaryl CoA (HMG-CoA), which is converted to mevalonate by HMG-CoA reductase (HMGR). The mevalonate is sequentially phosphorylated and decarboxylated to generate isopentenyl pyrophosphate (IPP). Condensation of dimethylallyl diphosphate (DMAPP) with one IPP generates geranyl diphosphate (GPP), and the addition of a second IPP unit generates farnesyl pyrophosphate (FPP). Squalene synthase (SS) catalyses the first enzymatic step from the central isoprenoid pathway toward sterol and triterpenoid biosynthesis [5]. Squalene epoxidase (SQE) catalyses the first oxygenation step in phytosterol and triterpenoid saponin biosynthesis. Both phytosterols and triterpenes in plants are synthesised from the product of OSC-catalysed cyclisation of 2,3-oxidosqualene.

It has been suggested that the HMGR, SS, and SQE enzymes of the mevalonate pathway represent the rate-limiting or regulatory enzymes for saponin biosynthesis [15]. The diverse triterpene skeletons are determined by OSC. All the genes encoding enzymes involved in the upstream regions of saponin biosynthesis were successfully identified in the leaf transcriptome of *E. senticosus* (Table 4). All transcripts were annotated by more than one unique sequence as the same enzyme. A putative sequence with high similarity to SQE was found to comprise the most abundant 17 unique sequences (Table 4). The OSC sequences with high similarity to β-amyrin synthase gave the greatest number of reads (Table 4).

**Table 4:** **Number of putative unique sequences involved in saponin skeleton biosynthesis**

**Enzyme code**	**Enzyme name**	**Number of unique sequences**	**Number of 454 reads**
2.3.1.9	Acetyl-CoA acetyltransferase	8	13
2.3.3.10	HMG-CoA synthase	8	84
1.1.1.34	HMG-CoA reductase	11	125
2.7.1.36	Mevalonate kinase	7	7
2.7.4.2	Phosphomevalonate kinase	1	8
4.1.1.33	Mevalonate-5-diphosphate decarboxylase	4	20
5.3.3.2	Isopentenyl-PP isomerase	1	22
2.5.1.10	Farnesyl diphosphate synthase	2	121
2.5.1.21	Squalene synthase	6	71
1.14.99.7	Squalene epoxidase	17	213
5.4.99.39	β-Amyrin synthase	4	323
5.4.99.41	Lupeol synthase	1	31
5.4.99.8	Cycloartenol synthase	10	36

### Oxidosqualene cyclase

**Figure 3:**
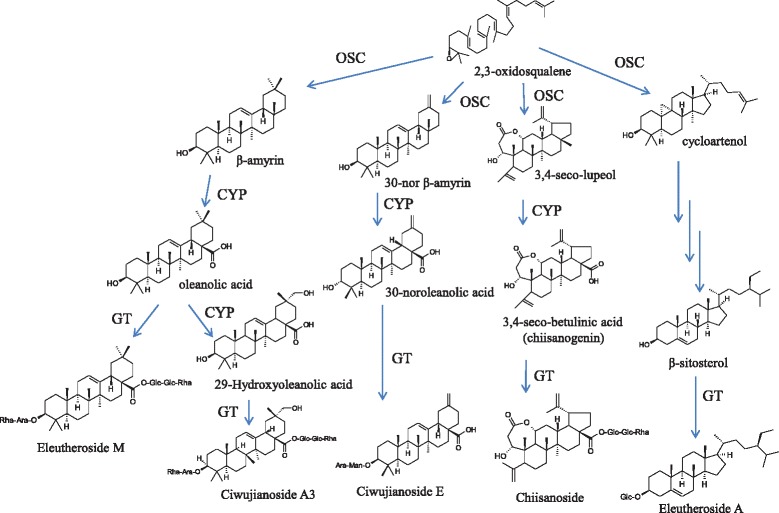
**Putative saponin biosynthetic pathway from 2,3-oxidosquane in** ***E. senticosus*** **.**

Triterpenes are one of the largest classes of plant metabolites and have important functions. A diverse array of triterpenoid skeletons are synthesised via the isoprenoid pathway by OSC. The major saponins in *E. senticosus* are eleutherosides I, K, L, and M and ciwujianosides A1, C3, C4, and D1*,* all these are called oleanane-type saponin derived from β-amyrin. We suggest that the aglycone of ciwujianoside E may be formed from 30-noroleanolic acid, which is frequently observed in natural compounds [16] and may be derived from 30-nor β-amyrin. The aglycone of chiisanoisde is reportedly 3,4-seco-betulinic acid (Chiisanogenin) [17]. Because betulinic acid is derived from lupeol, the triterpene precursor of 3,4-seco-betulinic acid may be 3,4-seco-lupeol. Thus, *E. senticosus* may have special types of OSC genes for the production of 30-nor β-amyrin and 3,4-seco-lupeol. In Figure 3, we propose that putative 4 OSC genes are involved in triterpene biosynthesis in *E. senticosus*.

**Figure 4:**
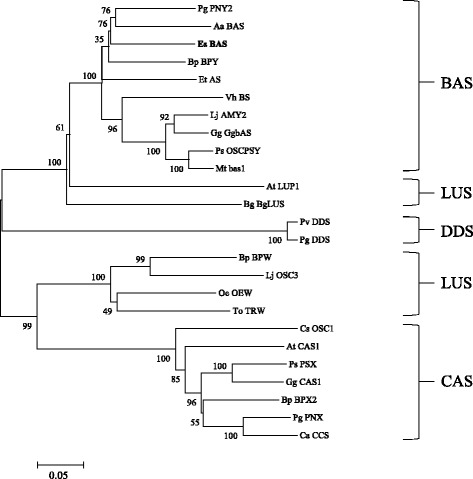
**Phylogenetic tree of the deduced amino acid sequences of EsBAS and other plant OSCs.** Phylogenetic trees of plant OSC distances between each clone and group were calculated using the program CLUSTAL W. The distance between each clone was calculated using CLUSTAL W. Bootstrap analysis values are shown at the nodal branches. The indicated scale represents 0.1 amino acid substitutions per site. Pg, *Panax ginseng*; Aa, *Artemisia annua*; Es, *Eleutherococcus senticosus*; Bp, *Betula platyphylla*; Et, *Euphorbia tirucalli*; Vh, *Vaccaria hispanica*; Lj, *Lotus japonicas*; Gg, *Glycyrrhiza glabra*; Ps, *Pisum sativum*; Mt, *Medicago truncatula;* At*, Arabidopsis thaliana;* Bg*, Bruguiera gymnorrhiza;* Pv*, Panax vietnamensis;* Oe*, Olea europaea;* To*, Taraxacum officinale;* Cs*, Crocus speciosus;* Ca*, Centella asiatica.*

The 454 pyrosequencing of *E. senticosus* revealed a total of 15 OSC sequences, among which 4 transcripts with 323 reads were putative β-amyrin synthases, 10 transcripts with 36 reads were cycloartenol synthases, and one transcript with 31 reads was a putative lupeol synthase. An OSC full sequence (*EsBAS*) with high similarity to β-amyrin synthase was obtained (Additional file 1). The *EsBAS* cDNA was 2,738 bp long and included a 2,295 bp full open reading frame (ORF) fragment. The deduced amino acid sequence of EsBAS (769 amino acids with a predicted molecular mass of 88.4 kDa) is 92% and 84% identical to β-amyrin synthase (PgPNY1) in *P. ginseng* and OSCBPY in *Betula platyphylla* (Figure 4). The relatively high identities of the *EsBAS* protein with other β-amyrin proteins suggest that this gene encodes a β-amyrin synthase in *E. senticosus*.

### Cytochrome P450s

CYP is a superfamily of monooxygenases, a large and diverse group of enzymes that catalyse the oxidation of organic substances. CYP is involved in a wide range of biosynthetic pathways, including those for lignin, terpenoids, sterol, fatty acids, and saponins [18,19]. In our sequencing results for *E. senticosus*, 84 contigs and 122 singletons were annotated as CYPs. These sequences were grouped into 32 CYP families with single and multiple copies (Additional file 2). The most abundant CYP transcripts (more than 500 454 sequencing reads) in the *E. senticosus* leaf belonged to the CYP72, CYP76, and CYP716 families. Of the 32 CYP families, we selected 22 CYP families with more than 40 copies of transcript reads as shown in Additional file 3. Among these 22 family sequences, 9 sequences had a full ORF region.

Based on the structure of the sapogenin aglycone, the non-saccharide portion of saponins, saponins from *E. senticosus* can be classified as several types of triterpenoid aglycones (oleanolic acid, 29-hydroxyoleanoic acid, 30-noroleanolic acid, and 3,4-seco-betulinic acid) and steroid aglycone (β-sitosterol), as shown in Figure 3. Thus, we propose that several CYP enzymes are involved in saponin biosynthesis in *E. senticosus*.

**Figure 5:**
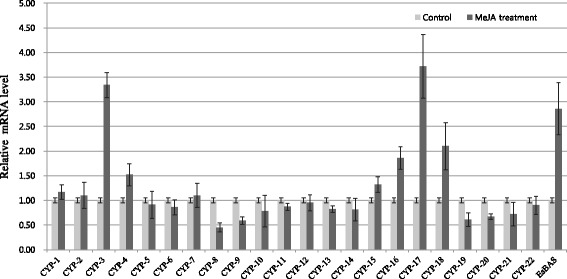
**qPCR analysis of 22 CYPs and** ***EsBAS*** **in MeJA-treated leaves of** ***E. senticosus*** **.** The relative fold expression of genes in MeJA-treated leaves and untreated controls is shown. *EsBAS*, putative β-amyrin synthase in *E. senticosus*.

Methyl jasmonate (MeJA), a type of elicitor, has been used to increase saponin production in plant cell culture [20]. MeJA treatment also induces the strong up-regulation of enzymes related to saponin metabolism [21]. To discover genes involved in saponin biosynthesis in *E. senticosus*, the transcription of 22 putative CYP genes in MeJA-treated leaves was monitored by qPCR for 1 day. Because the genes involved in the saponin biosynthetic pathway are simultaneously enhanced after MeJA treatment in many species, the putative β-amyrin gene (*EsBAS*) was used as a control to screen the putative CYP genes involved in saponin biosynthesis in *E. senticosus*. The transcription of the *EsBAS* gene was increased 3-fold after MeJA treatment compared to non-treatment. Three sequences of putative CYPs (CYP-3, CYP-17, and CYP-18) were clearly up-regulated by MeJA at least more than 2-fold (Figure 5).

**Figure 6:**
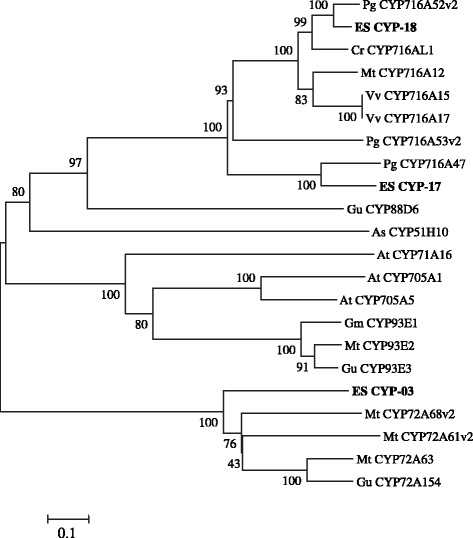
**Phylogenetic tree of the deduced amino acid sequences of EsCYP-03, 17, 18 and other plant CYPs.** Phylogenetic trees of plant OSC distances between each clone and group were calculated using the program CLUSTAL W. The distance between each clone was calculated using CLUSTAL W. Bootstrap analysis values are shown at the nodal branches. The indicated scale represents 0.1 amino acid substitutions per site. Pg, *Panax ginseng*; Es, *Eleutherococcus senticosus*; Cr*, Catharanthus roseus*; Mt, *Medicago truncatula*; *Vv, Vitis vinifera*; Gu, *Glycyrrhiza uralensis*; As, *Avena strigose*; At*, Arabidopsis thaliana*; *Gm, Glycine max.*

Phylogenetic analysis revealed that CYP-3 belongs to the CYP72A subfamily (Figure 6). In *Glycyrrhiza* (licorice), CYP72A154 catalyses C-30 oxidation of β-amyrin [22], and CYP72A61v2 and CYP72A68v2 in *Medicago truncatula* modify 24-OH-β-amyrin and oleanolic acid, respectively [23]. CYP-17 is similar to *P. ginseng* CYP716A47, which is dammarenediol 12-hydroxylase [24] (Han et al. 2011). The deduced amino acid sequence of CYP-17 is 49% homologous to CYP716A47.

The sapogenin structure of eleutherosides I, K, L, and M and ciwujianosides A1, C3, C4, and D1 in *E. senticosus* is oleanolic acid which is derived from β-amyrin (Figure 3). β-amyrin is converted to oleanolic acid after hydroxylation by CYP716A subfamily enzymes [23,25,26]. *CYP716A12* from *Medicago truncatula* and *CYP716A52v2* from *P. ginseng* are β-amyrin 28-oxidases (oleanolic acid synthases) belonging to the CYP85 clan [23,26]. The full sequences of the CYP-18 gene are 92% and 95% similar to *CYP716A52v2* from *P. ginseng* and *CYP716A12* from *M. truncatula*, respectively. Thus, we propose that the CYP-18 sequence is the best candidate CYP gene determining sapogenin formation in the biosynthesis of eleutherosides I, K, L, and M and ciwujianosides A1, C3, C4, and D1. The enzymes responsible for 29-hydroxyoleanolic acid formation from oleanolic acid, 30-noroleanolic acid formation from 30-nor β-amyrin, and 3,4-seco-betulinic acid formation from 3,4-seco-lupeol have not been identified. Thus, *E. senticosus* CYP enzymes and their effect on sapogenin aglycone formation merit further study*.*

### UDP-glycosyltransferases

Saponins are high molecular weight glycosides consisting of a sugar moiety linked to a triterpenoid or steroid aglycone. All saponins feature one or more sugar chains attached to the aglycone. Glycosylation contributes to the highly diverse nature of plant secondary metabolites. UGT is a superfamily of enzymes that catalyses the addition of the glycosyl group from a UTP-sugar to a sapogenin molecule. Thus, UGTs are important for the regulation of saponin biosynthesis. Normally, UGTs act at the last stage of natural plant secondary metabolites and have a significant role in the stability of products and modification of biological activity [27]. In this study, 144 unique UGT sequences were identified in the *E. senticosus* transcriptome. They were classified into 18 UGT families as shown in Additional file 4. The UGT85 family gene had the most reads, with 39 unique sequences and 309 reads. The UGT73 family had the second highest number of reads, including 4 subfamilies and 14 unique sequences.

**Figure 7:**
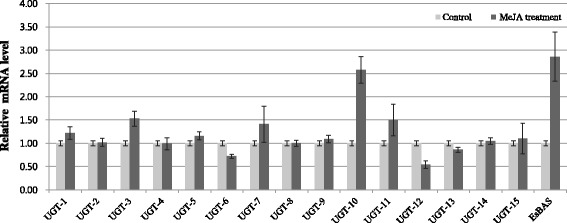
**qPCR analysis of 15 selected UGTs of** ***E. senticosus*** **in MeJA-treated materials.**

**Figure 8:**
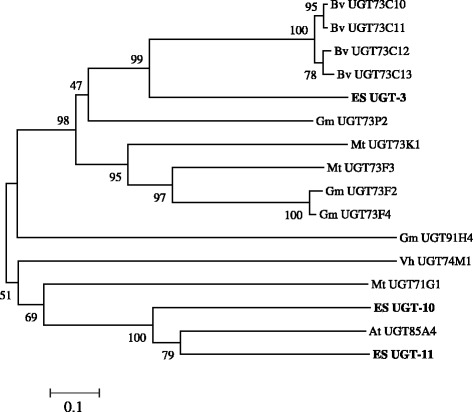
**Phylogenetic tree of the deduced amino acid sequences of EsUGT-3, 10, 11 and other plant UGTs.** Phylogenetic trees of plant OSC distances between each clone and group were calculated using the program CLUSTAL W. The distance between each clone was calculated using CLUSTAL W. Bootstrap analysis values are shown at the nodal branches. The indicated scale represents 0.1 amino acid substitutions per site. Bv, *Barbarea vulgaris*; Es, *Eleutherococcus senticosus*; *Gm, Glycine max*; Mt, *Medicago truncatula*; *Vh, Vaccaria hispanica*; At*, Arabidopsis thaliana.*

Fifteen unique sequences from each UGT family were screened to discover genes involved in saponin biosynthesis (Additional files 5 and 6). MeJA treatment also resulted in strong up-regulation of UGT enzymes related to saponin metabolism [21]. The transcription profiles of 15 UGT sequences were examined in MeJA-treated leaves of *E. senticosus* to screen the UGT genes involved in saponin biosynthesis. As shown in Figure 7, the expression of three UGT (UGT-3, UGT-10, and UGT-11) sequences was increased at least 1.5-fold after MeJA treatment. Transcription of *EsBAS* was increased approximately three-fold in MeJA-treated leaves compared to the control. The transcription of UGT-10 and UGT-11, which belong to the UGT85A subfamily, was enhanced approximately 2.5-fold in MeJA-treated leaves compared to the untreated control (Figure 7). The UGT-3 sequence belongs to the UGT73C subfamily (Figure 8). The involvement of UGT73 family genes in saponin glycosylation has been reported previously for other plants [28].

## Discussion

In the present study, transcriptomic analysis of *E. senticosus* leaves was performed using the GS FLX Titanium platform. A total of 15,217 contigs and 57,594 singletons were generated by assembling 627,923 reads. The most abundant cDNA sequences of the leaf transcriptome of *E. senticosus* were chloroplast-specific genes. However, we identified all sequences involved in the upstream region of the mevalonate pathway for saponin biosynthesis, from acetyl-CoA to SS, in *E. senticosus* by searching these transcripts against sequence databases using the blastX algorithm. *E. senticosus* leaves are rich in saponins. Of a total of 43 triterpenoid saponins in *E. senticosus,* 26 were isolated from leaves [3].

SQE enzymes catalyse the conversion of squalene to 2,3-oxidosqualene. In the transcriptomic analysis of *E. senticosus,* sequences encoding SQE represented the highest number (17) of unique sequences among transcripts associated with the mevalonate metabolic pathway, with 213 sequence reads. SQE is likely an important regulatory enzyme in this pathway [15]. Single copies of the SQE gene are found in yeast and mouse, and thus disruption of SQE in these organisms is lethal [29]. By contrast, plants examined thus far have two or more copies of SQE genes. In *Arabidopsis thaliana*, 6 SQE isoforms have been identified [30], of which *SQE1*, *SQE2*, and *SQE3* encode functional SQEs, while *SQE4*, *SQE5*, and *SQE6* fail to complement the yeast erg1 mutation. Rasbery et al. [30] suggested that *SQE* genes have different isoform-dependent functions in Arabidopsis. In *Medicago truncatula* cell cultures, the *SQE* gene *MtSQE2* was up-regulated by treatment with MeJA, while *MtSQE1* was not [31]. Han et al. [32] reported that the expression of *PgSQE1* and *PgSQE2* regulated in different manners and that *PgSQE1* regulates the biosynthesis of ginsenoside but not phytosterols in *P. ginseng*. The SQE gene responsible for saponin biosynthesis among the 17 unique SQE sequences in *E. senticosus* remains to be identified.

The cyclisation of 2, 3-oxidosqualene is a branch point of phytosterol and saponin synthesis, which play an important role in carbon flux regulation in other metabolic branches. In the transcriptomic analysis of *E. senticosus*, sequences encoding lupeol and cycloartenol synthase were represented in 31 and 35 reads, respectively. β-Amyrin synthase represented four unique sequences with 323 reads and thus had the most reads among the upstream genes of saponin biosynthesis (Table 4). This result suggests that transcriptional activity for oleanane-type saponin biosynthesis starting from β-amyrin may be very high in leaves of *E. senticosus*. Among the *Arabidopsis* OSC enzymes, *ATBAS* (AT1G78950) encodes a multifunctional OSC yielding more than nine products, including β-amyrin, α-amyrin and lupeol [33]. Tomato SlTTS1 (SlBAS) forms β-amyrin as its sole product, while SlTTS2 catalyses the formation of seven different triterpenoids, with δ-amyrin as the major product [34]. *E. senticosus* may produce 30-nor β-amyrin and 3,4-seco-lupeol, and the characterisation of the OSC genes involved in the biosynthesis of these triterpenes will be of interest in future work.

The cyclised triterpenes undergo two additional transformations (hydroxylation and glycosidation). In oleanan-type saponin biosynthesis, the oleanolic acid sapogenin is synthesised from β-amyrin after oxidation by CYP [23,26], and this sapogenin is further glycosylated by UGT to produce various type of saponins.

The major saponins of *E. senticosus* are the eleutherosides I, K, L, and M and the ciwujianosides A1, C3, C4, and D1, which are oleanane-type triterpenoids derived from β-amyrin triterpene. Among CYP ESTs, the sequence encoding the β-amyrin gene has the most abundant reads in *E. senticosus*. This result suggests that some CYPs and UGT genes involved in oleanane-type saponin biosynthetic pathway may be abundant in EST sequences of *E. senticosus. CYP716A12* in *M. truncatula* [25], *CYP716A16* and *CYP716A17* in *Vitis* [23], and *CYP716A52v2* in *P. ginseng* [26] have been identified as genes encoding β-amyrin 28-oxidase (oleanolic acid synthase). In the 454 dataset, we observed that CYP-18 of *E. senticosus* is highly homologous (90%) with *CYP716A52v2*. We suggest that this gene may convert β-amyrin to oleanolic acid in *E. senticosus*. Two other genes (CYP-3, CYP-17) that were increased by MeJA treatment compared to the control are also likely involved in saponin biosynthesis in *E. senticosus. CYP-17* has high similarity (86%) with *CYP716A47* from *P. ginseng,* which catalyses protopanaxadiol sapogenin formation from dammarenediol-II [24]. Fukushima et al. [23] reported that CYP716A subfamily members are multifunctional oxidases in triterpenoid biosynthesis. Thus, the *CYP-17* gene may be involved in saponin biosynthesis in *E. senticosus.* The *CYP-3* gene is 67% identical to *CYP72A63* from *M. truncatula* and 69% identical to *CYP72A154* from *Glycyrrhiza uralensis*. All three genes encode enzymes that catalyse β-amyrin oxidation to produce different types of aglycones in saponin biosynthesis [22].

UGTs involved in saponin biosynthesis belonging to the UGT 71, 73, 74, and 91 clans have been identified previously [28]. UGT73C10 to UGT73C13 in *Barbarea vulgaris* have been reported to be involved in C-3 glycosylation of hederagenin and oleanolic acid [35]. UGT73F2 and UGT73P2 from *Glycine max* catalyse the addition of Xyl and Glc, respectively, to the Ara residue at the C-22 position of soyasapogenol A [36]. Thus, the UGT 73 clan is the best candidate group for oleanane-type saponin biosynthesis. In this study, we identified a gene (UGT-3) belonging to the UGT 73 clan whose transcription was enhanced approximately 2-fold by MeJA treatment compared to the control. However, the UGT85 family of sequences (UGT-10 and UGT-11) exhibited the highest enhancement of transcription after MeJA treatment. Based on the most abundant transcripts in the *E. senticosus* transcriptome analysis, the UGT-10 and UGT-11 sequences belonging to the UGT85A subfamily are the best candidate genes for saponin biosynthesis in *E. senticosus.* As shown in Figure 3, the huge biodiversity of saponins in *E. senticosus* suggests that various UGT genes are involved in each specific step of saponin biosynthesis. Further characterisation of UGT family enzymes is needed to validate the pathway of saponin biosynthesis in *E. senticosus*.

## Conclusions

In this research, a large-scale EST sequencing was performed in leaf tissues from *E. senticosus*. The obtained EST dataset provides a useful information for gene discovery and genetic analysis in this plants. The genes involved in saponin biosynthesis pathway as well as candidate genes that might be involved in the triterpene formation, hydroxylation or oxidation of triterpene skeletons by CYP and glycosylation by UGT will help the further research for conducting the functional genomics and transcriptomics of *E. senticosus.*

## Methods

### Plant materials

Fresh leaves of *E. senticosus* were collected from Mt. Odae, Pyeongchang, Kangwon-do, Korea. To determine the effect of elicitor treatment on the transcriptional activities of specific genes, leaves were exposed to 200 μM MeJA for 8 h, and control leaves were treated with 0.25% ethanol. All tissues were immediately frozen in liquid nitrogen and stored at −80°C until use.

### RNA extraction

Total RNA was extracted from leaves using Trizol reagent (MRC, USA) and RNeasy® Plant Mini Kit (QIAGEN, Germany) according to the manufacturer’s instructions. Genomic DNA was removed from the total RNA using DNase following the manufacturer’s protocol (TAKARA, Japan). mRNA was isolated from 100 μg of total DNase-treated RNA using an mRNA purification kit (Stratagene, USA) according to the manufacturer’s instructions. Agarose gel electrophoresis and the OD260/280 ratio were used to assess the quality of RNA before cDNA synthesis.

### cDNA preparation and sequencing

mRNA was purified using poly-T oligo-attached magnetic beads and then fragmented with the RNA fragmentation solution supplied with the GS Titanium Library Preparation kit (454 Life Sciences, Branford, CT) following the manufacturer’s recommendations. The first- and second-strand cDNAs were synthesised and end-repaired. Adaptors were ligated at the 5′ and 3′ ends. cDNA libraries were validated using a High Sensitivity Chip on the Agilent2100 Bioanalyzer™ (Agilent Technologies, CA). emPCR reactions were performed on enriched cDNA templates.

The emulsions were broken, and the DNA capture beads were collected. The enriched bead samples were counted according to the manufacturer’s instructions (Roche). Tagged libraries were combined in a picotitre plate for sequencing. A one-plate reaction of 454 pyrosequencing was conducted using the Roche 454 Genome Sequencer FLX System (Branford, CT, USA).

### *De novo* assembly

The 454 Genome Sequencer FLX system collects the data and generates a standard flow gram file (.sff) that contains raw data for all the reads. The raw data were quality-filtered using a quality cut-off value of 40. The primer and adapter sequences that were incorporated during cDNA synthesis and normalisation were removed. Sequences of less than 50 bp were removed before contig assembly. De novo contig assembly of the reads was performed using GS De Novo Assembler software provided by 454 Life Sciences Corp, CT, USA. The assembly parameters were a minimum overlap length of 40 bp and a minimum overlap identity of 95%.

A total of 627,923 reads were assembled as 15,217 contigs and 57,594 singletons, which were functionally annotated using the BLASTN program. Putative protein-encoding sequences were compared with the databases KEGG (http://www.genome.jp/kegg/) and UniProt (http://www.uniprot.org/) and searched against the Nr (www.ncbi.nlm.nih.gov) database using the BLASTX algorithm with a cut-off E value of 10^−5^. The functional categories of these sequences were matched to the gene ontology (GO) algorithm.

### qPCR analysis

RNA was isolated from control and MeJA-treated leaves and reverse transcribed using the ImProm-II Reverse Transcription System (Promega, Madison, WI, USA). qPCR was performed using a Qiagen Rotor Gene Q Real-time PCR detector system with SYBR Green PCR Kit (Qiagen, Germany). Two-step amplification conditions for all real-time PCRs were 95°C for 5 min, followed by 40 cycles of 95°C for 5 sec and 60°C for 10 sec. The qPCR data are presented as the average relative quantities ± SE from at least three replicates. For the MeJA inducibility experiment, the expression of each gene was used as the calibrator. The relative expression value of each gene was calculated using the ^-ΔΔC^T method [37]. The *E. senticosus β-actin* gene was used for normalisation. All primers used in the present study are listed in Additional files 4 and 7.

### Phylogenetic analysis

The deduced amino acid sequences of the *EsBAS*, CYP and UGT genes of *E. senticosus* and those of other plants were obtained from DDBJ/GenBank/EMBL for phylogenetic analysis. Multiple sequence alignments were generated using the CLUSTAL W program [38]. Phylogenetic analysis was performed using the neighbour-joining method with the MEGA 5.0 software program [39]. A bootstrap of 1,000 replications was used to estimate the strength of nodes in the tree [40].

### Availability of supporting data

The transcriptome sequence data have been deposited into the NCBI Short Read Archive (SRA, http://www.ncbi.nlm.nih.gov/sra/) under the accession numbers SRR1611617. The phylogenic alignments have been deposited in TreeBase; submission ID 17087, (http://treebase.org/treebase-web/search/study/summary.html?id=17087&x-access-code=aef0b055f66288e54b73754f03fe0316).

## Additional files

Additional file 1:
**Sequence of beta-amyrin (** ***EsBAS*** **) gene in** ***E. senticosus.***
Additional file 2:
**Summary of family classification of the annotated CYPs from the 454 assembled unique sequences.**
Additional file 3:
**Lists of sequences of selected 22 CYP genes in** ***E. senticosus.***
Additional file 4:
**List of CYPs gene-specific primer sequences used for qPCR analysis.**
Additional file 5:
**Summary of family classification of the annotated UGTs.**
Additional file 6:
**Lists of sequences of selected 15 UGT genes in** ***E. senticosus.***
Additional file 7:
**List of UGTs gene-specific primer sequences used for qPCR analysis.**
