# Supplementary material for: Transcriptomic analysis of Siberian ginseng (Eleutherococcus senticosus) to discover genes involved in saponin biosynthesis
Source: BMC Genomics. 2015 Mar 14;16(1):180. doi: 10.1186/s12864-015-1357-z (PMC4369101; doi:10.1186/s12864-015-1357-z)
Supplement: Additional file 1: — Sequence of beta-amyrin ( EsBAS ) gene in E. senticosus. [file 12864_2015_1357_MOESM1_ESM.pdf]

Additional file 1: Sequence of beta-amyrin (*EsBAS*) gene in *Eleutherococcus senticosus*

>EsBAS

AATaATcAACAGTTTGAGAGtTAgAGtTTGTTAAtTTCTAtGCAAGATCGATCTaATTGGCTGCAAGAGCTCATCAACCAGCTGAAACTCTCAAAGATTGTGT
ACACTTTACAGCATCTCCGGCAGCGGTGGTAGAAATTGTGAGAATTGATAAACAAATAAATAAATCATGTGGAAGTTGAAGATAGCGGAAGGAGATGAA
AATGACCCGTATTTGTACAGCACCAATAATTTTGTCGGGCGGCAAACATGGGAGTTCGACCCGGATTATGTTGGTAGCCCGGGAGAGCTAGAGGAGG
TGGAAGAAGCTCGGCGTGAGTTTTGGGAGAACAGGCATAAGGTCAAGGCTTGTGGCGATCGCCTCTGGCGTATGCAGTTTCTAAGAGAGAAGAATTT
CAAACAAACAATCCCCCAAGTGAAGGTAGGAGATGACGAGGCAGTTACTTTTGAGGCCGCCACTACGACACTCCGAAGGGCTGTCCACTACTTTTCAG
CTGTGCAGGCCAGCGACGGTCATTGGCCTGCCGAGATTGCCGGACCTCTCTATTTCCTTCCGCCCTTGGTGATGTGTGTATATATCACAGGGCATCTT
GATACAGTGTTCCCAGCAGAACATCGAAAAGAAATTCTTCGCTACATATATTGTCATCAGAATGAAGATGGCGGGTGGGGATTCCATATTGAGGGGCAT
AGCATCATGTTCTGCACAGTTCTTAGCTACATTTGTATGCGTATACTTGGAGAAGGACCCGATGGTGGTGTAAACAATGCATGTGCCAGAGGCCGAAA
ATGGATCCTTGACCATGGCAGTGCAACCGCTATACCTTCATGGGGCAAGACTTGGCTTTCGATACTTGGTGTATATGAATGGACGGGAAGCAACCCAA
TGcCCCCAGAATTCTGGATTCTCCCTTCTTTCCTTCCTATGCACCCAGCTAAAATGTGGTGTTATTGCCGGATGGTTTACATGCCAATGTCATATTTATAT
GGGAAGAGGTTTGTTGGTCCAATCACTCCTCTCATTTTACAATTAAGAGAAGAACTATATGCTCAACCCTACAATGAAATCAAGTGGAGCAAAGTACGA
CATGTGTGCGCCAAGGAGGACATCTATTATCCTCACCCTTTAATACAAGACCTGATCTGGGATAGTCTCTATATATTAACTGAACCTCTTTTAACTCGTT
GGCCATTTAACAAGTTGAGAGAGAAAGCTCTGCAGACTTCCATGAAGCACATTCACTATGAAGATGAGAACAGTCGATATATTACCATTGGATGTGTGG
AAAAGGTTTTGTGTATGCTTGCTTGTTGGGTTGAGGATCCAAATGGAGATTATTTCAAGAAACACCTTGCAAGGATTCCAGATTATATATGGGTTGCTGA
AGATGGAATGAAGATGCAGAGTTTTGGTAGTCAGGAATGGGATACAGGTTTTGGCATTCAAGCATTGTTGGCTAGTGATCTCACTCATGAAATTGGACC
TACTCTTATGAAAGGACATGACTTCATCAAAGAGTCCCAGGTCAAGGATAATCCTTCTGGTGACTTCAAAAGCATGTATCGCCACATTTCCAAAGGATC
GTGGACTTTTTCAGATCAAGATCACGGATGGCAAGTTTCTGATTGTACTGCAGAAGGATTAAAGTGTTGCCTTATTTTCTCAACAATGCCAGAGGAAATC
GTTGGCAAGAAAATGGAACCCGAACTACTGTATAATTCTGTTAATGTATTGCTTTCCCTACAGAGCAAAAATGGTGGGTTAGCAGCATGGGAGCCTGTA
ACAGGTCAGGACTGGTTAGAGTTGCTCAATCCTACGGAATTCTTTGAAGACATTGTCATTGAGCACGAGTATGTAGAGTGCACTTCATCGGCAATTCAA
GCCCTGGTTCTGTTTAAAAAGGTATATCCTGGGCACCGAAAGAAGGAGATAGATAATTTTATTACGAATGCTATTCGTTACCTTGAAGACATACAAATGC
CTGATGGTTCATGGTATGGAAACTGGGGTGTGTGTTTTACTTACGGTAGCTGGTTTGCTCTTgGGGGGCTAGCGGCAGCTGGAAAGACATACTATAATT

GTGCAGCTGTTCGTAAAGCTGTTAATTTTCTGCTCGAATCACAGTTGGATGATGGCGGTTgGGGGGAAAGCTATCTTTCTTGCCCGAAAAAGGTATATG
TACCATTAGAAGGAAACCGCTCAAATTTGGTGCATACTGCATGGGCCTTAATGGGACTGATTCATTCTGGGCAGGCCACGAGAGACCCAACACCTCTT
CACCGTGCAGCCAAGTTATTGATCAATTCCCAGATGGAAGATGGTGATTTTCCCCAACAGGAATTAACTGGAGTTTTTATGAGGAATTGCATGTTGCAC
TATACAAATTACCGGAATGTATACCCATTGTGGGCTCTGGCAGAGTATCGGAGGCAGGTCCCATTACCGTCCCAGGCGCCTAATTAGCTATCGGGCTC
TTGGTAATTCAGTGCTAAATAATTTCAATAAAAGTAAAAGTGAGCATTGTTTTATTTAGTTGGTCTGTTGCAACTCCAGAGGCCCCTAAATAATATCAATT
CGTGGAATCACGCAGTACTGTTGAATGACTATTATTGCCTCTTGTTCATTTGATCTTGTATCATCATCCATATAATGGTTTGATTCACCTCATTGTACACA
ACATGCAACaAATTTTGGAGTTTCAtAAATGGTGTACACCTTAAATAGTCTTACTACATAA
