# Supplementary material for: Transcriptomic analysis of Siberian ginseng (Eleutherococcus senticosus) to discover genes involved in saponin biosynthesis
Source: BMC Genomics. 2015 Mar 14;16(1):180. doi: 10.1186/s12864-015-1357-z (PMC4369101; doi:10.1186/s12864-015-1357-z)
Supplement: Additional file 2: — Summary of family classification of the annotated CYPs from the 454 assembled unique sequences. [file 12864_2015_1357_MOESM2_ESM.pdf]

Additional file 2: Summary of family classification of the annotated CYPs from the 454 assembled unique sequences.

CYP450 family	Subfamily No.	Unique gene No.	454 reads No.	Number	ORF region	Length	Homology to [species name]	Accession No.	E-value
CYP71	2	27	243	CYP-1	Full	1694	CYP71D313 [Panax ginseng]	JN604541	0.0
CYP72	2	10	540	CYP-2	Full	1735	CYP72A129 [Panax ginseng]	AEY75218	0.0
				CYP-3	Partial	758	CYP72A57 [Nicotiana tabacum]	DQ350355	3E-86
CYP73	1	5	55						
CYP74	1	1	80	CYP-4	Full	1616	CYP74B4 [Medicago sativa]	AJ249245	0.0
CYP76	2	12	547	CYP-5	Full	1725	CYP76A2 [Vitis vinifera]	XM_002283736	0.0
CYP77	1	1	73	CYP-6	Partial	1704	CYP77A2 [Vitis vinifera]	XM_002270700	0.0
CYP78	1	7	40	CYP-7	Partial	1492	CYP78A3 [Vitis vinifera]	XM_002266457	0.0
CYP81	1	4	11						
CYP82	5	20	100	CYP-8	Full	1840	CYP82C4 [Vitis vinifera]	XM_002284774	0.0
CYP83	1	2	2						
CYP84	1	5	5						
CYP85	1	6	35						
CYP86	2	12	230	CYP-9	Full	2334	CYP86A2 [Vitisvinifera]	XM_002275770	0.0
				CYP-10	Full	1874	CYP86B1 [Vitisvinifera]	XM_002282149	0.0
CYP87	1	2	2						
CYP89	1	4	22						
CYP90	3	6	41	CYP-11	Partial	1406	CYP90A2 [Camellia japonica]	AAZ39038	0.0
CYP92	1	3	84	CYP-12	Partial	1429	CYP92A2 [Nicotiana tabacum]	DQ350324	0.0
CYP93	1	1	1						
CYP94	2	7	41	CYP-13	Partial	956	CYP94A1 [Cucumis sativus]	XM_006481967	5E-139
CYP97	3	3	73	CYP-14	Partial	1807	CYP97A3 [Daucus carota]	JQ655297	0.0
CYP98	1	2	114	CYP-15	Partial	1613	CYP98A2 [Solanum tuberosum]	XM_006364715	0.0
CYP701	1	1	12						
CYP704	2	11	23						
CYP707	1	5	5						
CYP710	1	1	21	CYP-16	Partial	1334	CYP710A15 [Medicago truncatula]	DQ335802	0.0
CYP712	1	3	3						
CYP716	2	14	528	CYP-17	Partial	1495	CYP716A47 [Panax ginseng]	AEY75212	0.0
				CYP-18	Full	1721	CYP716A52v2 [Panax ginseng]	AFO63032	0.0
				CYP-19	Full	1685	CYP716B2 [Vitis vinifera]	XM_006340018	1E-159
CYP724	1	1	1						
CYP734	1	12	33	CYP-20	Partial	865	CYP734A1 [Glycine max]	XP_003526785	1E-99
CYP735	1	2	2						
CYP736	1	9	69	CYP-21	Partial	1357	CYP736A12 [Panax ginseng]	AEY75215	5E-84
CYP749	1	7	27	CYP-22	Partial	931	CYP749A20 [Panax ginseng]	AEY75214	3E-155
Total	47	206	3063						
