# Supplementary material for: Transcriptomic analysis of Siberian ginseng (Eleutherococcus senticosus) to discover genes involved in saponin biosynthesis
Source: BMC Genomics. 2015 Mar 14;16(1):180. doi: 10.1186/s12864-015-1357-z (PMC4369101; doi:10.1186/s12864-015-1357-z)
Supplement: Additional file 3: — Lists of sequences of selected 22 CYP genes in E. senticosus. [file 12864_2015_1357_MOESM3_ESM.pdf]

Additional file 3: Lists of sequences of selected 22 CYP genes in *E. senticosus*

>CYP-1

GAATTAATTCCAATGGAGCTTCAATTTTCCTCCTTCTCTGTTATAACTATTTCCTTAATTATTGTCTCCCTGTTCCTCTCCAAGCTCTTCAA
AAGATCCAAACCCGATAATCTTCCCCCGGGTCCGGCCAAGCTGCCGATCATCGGAAATTTGCTCCAACTGGCACGTGTCGACCCGGT
ACCGCATCGGGGTCTCCTTGAATTGGCTCAGAAATATGGACCATTGATGCACTTGCAGCTGGGCAAAATCTCGACAATTGTGGTGTCG
TCGCCGCGTGTGGCGAAAGAGGTGCTAAAAACGCATGACGTTTCTTGTGCGGATAGGCCTGATATGTTACTGGGCAGGATTATGTTGA
AGAATAGTAGGGACATAGTTCTGGCTCCCTATGGGGATTATTGGAGGCAAATGCGAAAAATCTCGACGACGGAGCTTCTAAGTGCGAAT
AAAGTCCGGTCGTTTCGCAATATTCGAGAGGAGGAATCGTGGCAAGTGGTGGAGTCCGTCAGGGCATCGGTGGGATCGCCGGTTAAT
TTTAGCACCCAAATTACGGGGATGGCCAACTCGGTGATTTGTAGGGCGGCGATCGGGAAGAAATGCGCGTATCAAGATGAGCTGATTG
AGGTGGTGGAAGATATTGCCTATTGGGGTTCGGGTCTTTTTATGGCGGATTTGTTTCCGTATTTGAAATTTCTCGAGTACGTCACGGGG
ATGAGGCCTAAATTGGAGAAGATGAGGCGAAAGCTAGATCATATATTTGATAATATTATTCAAGAGCACATGGAAAAGATGGCGAGCAAG
AAAGAGGGGAAAGTAACTGATGATGAGGAGGAAGATCTGATTGATGTTCTTCTGAGAATCAACCAGGGTCGAAGCCTGGAGATTCCCA
TCACCTCCAAGGACATCCAAGGCATCACTCTTGATATGTTCACAGCCGGAACTGACAGTACTGCAGCGACACTTCAATGGATAATGTCC
GAGTTGATGAGAAACCCCCAAGTGATGAAAAAGGCTCAAGCAGAAGTGAGAGAAGCTCTCAAGGGAAAGACGGTAATCCACGAAGCC
GACATTCAAGGATTGACTTACCTAAAACTCGTGATCAAAGAAACTCTACGCCTGCACGCACCCGTTCCCTTGTTAGTCCCCAGAGAATG
CAGGAAGCAATGTGAGATTGACGGATACACCATACCGGTAGGAACAAAAATCATGGTCAACGCTTGGGCGATTGGAAGGGATCCGGA
ATATTGGGTTGACGCAGAAAGTTTCGTACCGGAGCGATTTGGGAGTGCCTCTGTGGACTACATAGGAGCCAACTTTGAGTATATTCCAT
TCGGCGCAGGAAGAAGAATATGCGCAGGTATTGCATTTGCGGCCGCCACTCTTGAGCTTCCAATGGCTCAGTTACTCTATTACTTTGAC
TGGAAACTCCCTAATGACATGAAACCGGAAGATCTAGATATGGAAGAGACAATTGGAGCCACAGCTACAAGAAAAATAGCTTGATTTTG
ATTCCAACTTTACACACTCCAAGCCAAGGTTTAGAGTAATCAATAATGTATCACAATCTGAAATTTTTACGTATCGTGTAATAATGTCTAAAT
CTTTACTTGTAGGATATTGGTATGCAAGTTAAATAAGAACGATTAAATGTAATGTTATTACATTGAACTCTTAATTATTGAGAGATGATGTTT
ACG

>CYP-2

ATATCATCTGCCCACTCCTCTCTACTCTCTACTCTCTCTCGTTGATGGATTTAATTTTGAATCTGGTAGTAAGCTGCTGTGCAATTGTTGT
AGTGATATTTGGATGGAGAATCTTCAACTTTTTGTGGTTGAGACCAAAGAAGCTTGAAAAGATTCTGAGGCAGCAGGGTTTCGATGGAA
ATTCGTACAGGCTCTTGTTTGGAGACCTGAAAGATACAGCAGAGATAAGAAAAGAAGCCAAGTCCAAACCCATCAATCTTTGTGATGAT
ATTCTCCCACGCGCTGCCCCCGTAATCCACAATACCTTCAAAAAATATGGAAAAGATTCGTTTGTATGGCTTGGACCAAAACCATTGGT
GTACATCACGGACCCTGATGATATAAAAGAGGTGTTGAATAAGTTTTATCAGTTCCAGAAGCCCAGGGGAGGTAATCCTTTGACAAAATT
GCTTGCAACTGGACTACTAGATGCTGAGGGTGATAGATGGGTCAAACACAGGAAAATCATCAATCCTGCTTTCCATCTTGAGAAGTTGA
AGAATATGCTGCCAGCCATTTATTTGAGTTTTAGCGAGATCATGAGCAAATGGGAAGAAATGGTTTTGACAAAGGGGCGGTGTGAGTTG
GATGTATGGCCTTATTTAGAAACTTTTACCAGCGACGTCATTTCACGCACAGCATTTGGCAGTAGCTATGAAGAAGGAAGAAGGATATTT
CAACTGCAGAAGGAGCAAGCTGAGCTAGTATTACAGGCTTCACAAACAATTTACATCTCTGGAATGAGATTTTTACCAACTACAAGAAAC
AAGAGGATGAAGGAAATTAACAAAGAAGTGAAAGCTGCACTAAGGGGTATCATCAGCAAGCGGTTGAAGGGAATGGAAGCAGGAGAA
TGTAGTCATGATGACTTATTGGGTATATTATTGGATTCCAATTCTAAAGAAATTAAACAACATGGAAGTAAAGATTTTGGAATGACTATAGA
CGAGGTTATCGAAGAGTGTAAGTTATTCTATTTTGCTGGACAAGAGACAACCTCAAATTTGCTTGTGTGGACTATGATTTTATTGAGTCA
ACATCCACACTGGCAAGAAAGCGCTAGAGAAGAGGTTTTGTTGGCCTTTGGAAACAACAAACCGGATCTTGATGGGTTGAATCACCTC
AAAGTTGTAAATATGATTTTGCTGGAGGTTCTTAGATTATACCCGCCCGGTGTTACTCTTGGTCGAACTATATATGAAGAAATTAAATTAGG
ACAAAAATCTCTTCCTGCTGGAGTGCTTATACAGTTACCCATCATCATATTGCATCACGATCAAGAAATCTGGGGCGACGATGCTAAGGA
GTTCAATCCAGAGAGATTTTCTGAAGGCGTGCTGAAGGTAACGAAAGGGAAAGTCGTATATTTTCCTTTTAGTTGGGGTCCCCGAATTT
GCGTTGGACAAAATTTTGCAATGCTAGAAGCTAAAATGGCGTTGGCGATGATTTTACAGCGCTTTTCCTTTGTGCTTTCACCATCGTATA
CACATGCTCCCCATCCCATTTTAACTCTGCAACCTCAATATGGTGCACACTTGGTTTTGCAAAAACTAGCGTGTTAAATTGTAATGCTAG
GGATCAATAAAGATTACATGCGCGCTTACTAGTCTAGAATTCAGACATACTGTCTTGTGGTATGTGCTTGATTATTATGGTATGTAAGTTAC
CTTTTGTACAAGATTGTCGAAGACTAGG

>CYP-3

GTTAAATTGAACGACGATAATGGATGGAGTAGTAGTGTACAGCTCAATTGTATTGGGTGTTGGGATTGTAGTAGGGGTTTGGTGGTTT
TTGAACTGGGTGTGGGTGAGGCCGAAGAAGATGGAGAAGTTGCTCAGAAAACAAGGCTTTAAAGGAAACCCGTATCGGGTCTGGTA
CGGAGATTTGAGACAGCAGACTTCCATGGTCGAAGAAGCAATGTCTAAGCCCATCAATATCTCCGATGATATCGTGCCTCGGGTCAT
ACCCTTTGTGTCTCATCATGTCGATTGTTATGGTAAAAAATCTTTTATATGGAATGGACCGACAGCAAATGTGGTAATTGTGGATCCTG
AAATGGTAAAAGAAGTTTTTACGAAGAATTTTGTATTTCAAAAGCCGACGGCAAACCCGCTATTCAGGATGTTTTTAATAGGACTCCCA
TCAATTGAAGGAGAAAAGTGGGCTAAGCATAGAAGACTTCTCAAGCCTGCTTTCCTTATGGAAAGGCTGAAGGATATGCTGCCAGCC
TTTGATTTGTGTTGCTGTAAGATGACAACAAAATGGCAGCAGAAGATGTTGGAAGGGAATTCATGTGAGTTGGATGTGATGCCATATC
TCCAAACGTTAACTAGTGACGTGATTTCGCATACAGCATTTGGCAGTAGCTATGAAGAAGGAAGTCGGGTATTTGAACTTCAAATAGA
ACAACTTCAACTCGCAATTGAGACTTTGCAATCAGTTTACATTCCTGGATCGAGGT

>CYP-4
AAAAAAAAAATCATCTCTACAATAATGATGCTCAAAATGATGAGCAGCACAACCCCATCGCCGCCATCGCTTACACCGGCATCGACACC
AACACCACCGACATCTTTGCCGGTCCGTACAATTCCCGGCGGGTACGGGTGGCCGTTGCTGGGACCGATCTCGGACCGGCTATCGTA
TTTTTGGTTCCAGGGACCGGAGACATTTTTCAGGAACAAAATTGAGCAGAACAAGAGCACGGTTTTTCGGACTAATATTCCTCCGACGT
TCCCCTTCTTTGGGGTGAATCCGAACGTGATAGCGGTGCTGGACTGCAAATCATTCTCTCACTTATTCGACATGGAGTTGGTGGAGAA
AAAGAACACTCTGGTCGGAGATTTCATGCCGAGTACAAGCTTCACCGGCGATTTACGTGTGTGTGCATACCTTGATTCTTCTGAACCTA
AACACTCCCAGATAAAGAAGTTTGCAATGGATCTCCTAAAACGAAGCTCCAGCATATGGGTTCCCACCGTCACCTCCAACCTCGACAC
CATGTGGGACTCCATTGAATCCGACATCGCCAAATCCGGCTCCGCCTCCCCGCTCATCCCTCTCCAAAAGTTCATTTTCAACTTCCTAA
CTCGCTGCCTCGTTGGAGTTGACCCGTCCATATCCCCTGAAGTCGCCGAGTGTGGCTACACTTGGCTCGACATATGGCTCGCACTTC
AACTCCTCCCCACCGTCAAAATCGGCTTTCTTCAACCCCTCGAAGAGATCCTCTTCCACTCATTCTCTTACCCTTCTTTCCTAGTCACC
GGAGGCTACGATAAACTCACAAAGTTTATCGAGAAAGAAGCTCAAGAAGTGCTAAACCGAGGACAAACCGAGTACGGACTCACTAAAC
AAGAAGCCCTTCAAAATCTCTTGTTCATTCTCGGATTCAACGCGTTTGGCGGATTCTCCATTTTTTTCCCTTCTGTTGTTAGTGCCCTCG
GGTCGGATACAATCTTACAAGAGAAACTGAGAAAAGAGGTGAGAGAAATAACCGGAACGAGTCCTTTAACTTTTGACTCGGTGAAAGA

GTTAGAACTGGTTAACTCGTTTGTATACGAAACGCTCCGGCTCAACCCGCCGGTACCGTTACAATTCGCCCGGGCGAGGAAGGATTTT
AAACTGAGTTCGCATGACTCGGCGTTTGAGATAAAGAAAGGTGAGCTTCTTTGCGGGTATCAGCCGTTGGTGATGAGAGATGGGAAG
GTATTTGACAACCCGGAGAAGTTTGTATGGGATCGTTTTACGAAGGAGAAAGGGCGGGAATTACTGAGTTATTTGTTCTGGTCGAACG
GGCCGCAGACCGGGTCACCCAGCGAGTCGAACAAGCAGTGTCCTGCTAAGGATTATGTGACGCTTACTGCATCAATCTTTGTGGCTC
ATTTGTTTAAGAGGTATGATTCGTTTACGGTTGATTCTTCTGGATTGATCACGGCCGTTGAAAAGGCTAAATGAGAGTGTGATTGAAGAA
AAATAATATGTAATGTATGTATATGTGGATTTTAATTAATCTATGTGAGAGTTGTTTGTATGTATGTGTTGAGATTGAGATATGATATTTAATCA
AAAAGATGATTTTACTAGGG

>CYP-5
GTCAAACGTGAGAAATGGAGAGTAGTAATAGTTCACTTACCTGGCTGAGTGCTTATTTTTCAGCTGCTATTCTTACTATTCTTCTGCTC
AAATGGATAAACACCAGGAATAGCTCAACTAAACAGAAACCACCAGGTCCGCCAGCATGGCCAGTGGTTGGCAACATGTTCGACCTT
GGAACCATGCCACACCAGAACTTATACAAACTCCGATTCAAATACGGACCTGTTCTTTGGCTGAAGCTCGGTGTAGTTAATACCATGG
TTATACAGTCGGCCAAGGCCGCGGAAGAATTTTTCAAGAAACACGACGTTCAATTTTCTGACCGTAAGATTCCTGACGCACTGACGG
CCCTGAGCTACAACCAAGGATCATTAGCTTTCGGTAATTATGGTGCATATTGGCGCGTACTACGAAAGCTCTGCTCCATGGAGCTCCT
AGTCAACAAACGACTGGACGATTCCACTGAGATCCGGGAAAAGTGCATTGAGAAAATGGTCCGGTGGATTGAGGAGGATGCAGCAG
CCTCACGAGCTCAGGGAAGACCAGGCGAGGTGGAACTGCCCCACTTCCTCTTTCTCATGACTTTCAACCTGGTTGGTAACCTCATGT
TGTCAAGAGATCTCCTAGACTTGCAATCCAAGGAAGGGAAAGAGTTCTACAATGCAATGAGCAAGATCGTGGGGTGGATTGGGAAGC
CAAACGTAGCAGACTTCCTGCCGTTCTTGAAGTGGATTGATCCACAGAGGATTAAAACAAATATGGTGAAAGACATGGGAATAGCCAT
AAATATCGTAGCCAGCTTTGTGAATGACAGGATTCGGGAAAATATATCAAGCAGCGAGAAGGCGTCGAAGGATTTCCTGGATATGCT
GTTGGAGTATGAAGGTGACGGAAAAGAAGGGCCTGACAAAATTGAAGAGAAGAACATAATTATCATCGTACTGGAAATGTTTTTTGCT
GGGTCAGAAACTACAAGCAGCTCCATTGAATGGGCAATGGCAGAGCTATTACGCCACCCTGAATCGATGAGAAAGGTTAAAGAAGAG
CTCGATAGTGTTCTCGGACGAAGCAGAAATATCAAAGAGAGTGATATCGACAAACTGCCATATTTGCAGTCCGTGGTGAAGGAAACA
CTGAGGTTACATCCTGCAGCCCCTCTACTGCTTCCGCGAAATGCAATGCAAGATACCAACTACATGGGGTATTTCATACCCAAAAGTA

CACAAGTTTTTGTGAATGCATGGGCAATTGGAAGGGACCCAGATGCTTGGGATGACCCCTTGTCTTTCAAGCCTGAGAGATTTTCAG
GCATAAATACCGAGTACAAAGGGCAACACTTTGAATTAATTCCATTTGGATCTGGGAGACGGATTTGCGTAGGCATTTCCTTGGCCCA
CCGAATGGTTCACCTCACTTTGGCCACCTTGCTACGGTCCTTTGACTGGGAGCTTGATAGCTCTGTCACTCCAGAGACTCTGGACAT
GAAGGAGAGGCCGGGAATAACACTGCGGAAGTTTGAACCTCTGAAAGCAATACCCAAAAAACGAATCGTGCAAGGTTGAAATGATCC
TTTAAGACAGGAGTTTGTTCGACCTTTAGTTGGATTTACAGTTATTATGTTGTTCATCTTTTTGTATGGTTTTCAGGTAGACGAAAAGGG
TAGTTTCCTTAATAATCTAAATAATTTGCCTATGCAATTTTGAAATTATTGAAAATTTACAT

>CYP-6
TTATCGGGTCTAATCTTCTTGTTGACCCGCAAAACAAACGCAAAGAGGATCCGTCTTCCTCCAGGTCCACCGGGCTGGCCTGTGGTTG
GCAATCTGTTTCAAGTTGCCAACTCGGGCAAACCCTTCTTTCAGTACATTCGAGATCAATTGATACCCAAATATGGCCCGATACTGACTC
TCAAAATGGGTACCCGAACCATGATTATTATTAGTAGTGCAGAATTAGCCCATGAAGCGTTGATTGAAAAGGGTCAAGTTTTTGCTAGCC
GGCCCACAGAAAACCCGACCCGAACCGTTTTCAGCTGCAATAAGTTCACGGTCAACGCCTCCTTGTACGGCCCGGTTTGGCGGTCCC
TGCGGCGAAACATGGTTCAGAACATGCTTAGCGCCACTAGGCTTCGAGGATTCAGTGACGTCAGAGACGGCGCAATGGATAAACTCG
TTGACCGGCTTAAGGCAGAGGCCGCGGCTAACGGCGGCGCTGTTTGCGTGTTGAAAAATGCCCGGTTTGCGTTCTTTTGTATACTTTT
GACTATGTGTTTTGGGATTGAAATGGAGGAAGAAATGATCGAAAGGGTGGATCATATGATGAAGACGGTGTTGATTACTCTTGATCCGA
GGTTGGATGATTATTTACCACTTTTGAGCCCTTTTTTCTCCAAGCAAAGGAAGAGAGCAAGCCAAGTTCGAAAAGAACAAATCGAAACC
CTAGTTCCATTAATCAATCGGCGTCGTTTAGCGCTTGAAAATCCAGGATCGGATAAAACCGCAACGTCCTTTTCGTATCTAGACACGCTT
TTCGAACTTCAAATCGAAGGTCGAAAAGCAACTCCTAGCAATGATGAGATTGTCACGCTTTGTTCGGAATTTCTCAATGGCGGCACTGA
CACGACCGGTACTGCCATTGAGTGGGCCATAGCCCGATTTATTGATAATCCTGACATCCAAACCAAGCTTTACGAGGAAATCAAATCAAT
AGCGGGGGACAGAAAAGTTGATGAGAATGATTTACAAAAAATGCCCTACTTGAATGCCGTGTCAAAGGAGTTATTACGAAAACACCCTC
CTACGTACTTTTCACTTACCCATGCAGTTATTGAGCCGGCAAAGTTGGCGGGTTATGATGTACCTACTGATACAAATGTGGAGTTTTTCT
TACCAGGTATTTCGGAGGATCCGAAAATATGGTCCGACCCTGAAAAGTTCGACCCGGAGAGGTTTCTGACCGGCCGGGAGAACGCCG
ACATAACCGGGGTGACCGGAGTGAAGATGATACCGTTTGGGGTCGGGCGGAGGATTTGCCCTGGGTTAGCGATGGCTACAGTCCAC

GTAAATTTGATGATAGCAAGAATGGTTCAAGAATTCGAGTGGAGCGCTTACCCGGAAAATAGCAAGGTGGATTTTAGTGAGAAGTTGGA
ATTCACTGTGGTGATGAAGAATAGTCTAAGAGCCAAGATCAAGCCAAGGGTTTAAAAATATGCTTAAACTAATATGATGATATATGATAGTT
GCTTTTTTTTATTTTTTATATATGCATATTTTCATATTTTAATGCATGGTGGTTTAAATGTGTTTTGTTGATTTTGCTAGGAAATATATTGGCTA
TATTCATGAAAAATAAATGTTTTTTTTTGGCTGAATAGACGCTATCATGAATAATTACAAATTCAGGGTCATTTAGCCATTGTGTTTACTCG
GCTATGT

>CYP-7
TTCTTCTTATTTTGCTGATATGCCCACCATACCAGGTCCAAAGGGTGTTCCTTTTATAGGGAGCATGAGTCTCATGGCTGGTTTAGCCCA
CCACAAGATTGCAGCCATGGCTACAGCTTGTGGAGCCAACCGGCTCATGGCTTTTAGCCTTGGTGAAACAAGAGTCGTCGTTACATGT
AATCCAGATGTGGCCAAAGAGATTCTCAACAGCTCCGTCTTTGCTGATCGACCCGTCAAAGAATCGGCTTACAGCTTAATGTTCAACAG
AGCTATCGGCTTCGCTCCTTATGGCGTTTACTGGCGAACTCTTAGGAGAATTGCAGCGGCTCATTTGTTCTGTCCCAAGCAAATCAAAG
CCTCCGAGGCTCAAAGGTTCGATATTGCCCGTCAAATGGCTGCAATGTTTACTGGTGGTACGACGATCAATGTGCGGGAGGCCTTAAA
AACGGCGTCTCGTAATAATATGATGTGCTCTGTATTTGGACGAAAATATGGGCTCGATTCCAGAAACACCGAAACTGAAGAGCTAAGAA
AGCTAGTTGATGAAGGATATGAAATTTTGGGTACACTCAACTGGTCCGACCACCTCCCTTGGTTATCCGGTTTTGACCCACAGAAAATC
CGGGTCAGGTGCTCCAATCTGGTCCTTAAGGTGAACCGGTTCGTCAGCAGGATTATCTCTGAACACCGTGCTGATCAGACGGGTGGG
CTCATGAACCGCGATTTTGTGGACGTTTTACTCTCTCTTGGAGGTCGTGAACAACTATCAGACTCGGACATGATTGCTGTGCTTTGGGA
GATGATATTCAGAGGAACTGATACGGTGGCGGTTTTGATGGAGTGGGTGCTAGCCAGGATGGTGCTTCATCCTGATGTTCAATCAAGG
GTCCATGATGAATTGGATACGATAGTTGGGAGGTCACGTGCGGTGACGGAGGCCGATATTGCAACCATGGTGTATTTACCAGCAGTTG
TAAAAGAAGTACTAAGGCTACACCCGCCGGGGCCATTGCTGTCGTGGGCCCGGCTGGCAATTACGGACACTACCGTGGATGGCTATC
ATGTTCCGGCCGGGACCACCGCGATGGTGAACATGTGGGCCATTACAAGGGACCCACGCGTGTGGACAGACCCACTGGCATTTAAG
CCGGAGAGGTTCGTCAATGAGGCCGCCGAGGGGATGGAGTTCTCAGTGATGGGGTCTGACCTTAGGCTGGCACCATTCGGGTCCGG
CAGGCGGACATGTCCCGGTAAAGCACTAGGGTTGACCACGGTCAACTTTTGGGTGGCATCGCTCTTGCACGAGTTCAAGTGGGGTG
GTTCAGGGCATTTGTCGGAGGTGCTAAAACTTTCGTGCGAGATGGCTAACCCACTCGTAGCTAAGGTGCAGCCAAGGCGCACGTCAA

GTTTAGCCCGTTAGGCTGGCTTGAAGCAAAACAGTGGAGTGGAGTGGAGTAGTGATGAAGAAAAGGAAGCGAAAAGAAATGTGCAG

>CYP-8

TCCTTTTCTAGTTAGCGTACGTCTTATGATAGACGGGGAAGAGTGATCTCTAGTTTAAGGAATGGCATTCTACAATCAGCTGCAAGAC
ATCACACTGCTCGGCCTTCTTTTTGCTGTTATTTGTCTATGGAAGATCATCAGTACTCATGCCAGAAGCAACAAGAGCAGTTCCAGAC
CTCCAGAACCTGCTGGGGCATGGCCTGTTATTGGTCACCTTCATCTTCTAGGAGCAAACAAGATATTACATCATATTCTTGGAGACAT
GGCGGATAAATATGGACCTATCTTCTCTTTGCAACTGGGGATTCACAGCACACTTGTGGTCAGTAACTGGGAAGTTGCAAAAGAGTG
CCTTACAACCCAGGACAAAGTGTTCTCTAACCGTCCAAAATCTCTAGCCATAAAGATCATGGGCTATGGCAATACCATCATTGGATTT
CTGCCTTATGGTCCATACTGGCGTGATATACGTAAGCTAGCAATAGTTGAGCTTCTATCCACCCGGCGACTTGATGTGCTCAAGCATG
TTCGGGAATCAGAGGTCAATAGTTTCGTCAGGGAGCTGTATGAGCAATGTATAAGCAATGAAGGATGTGGAAGCAAAGTTGTGGTGG
AAATGAAACAGAGGTTTGGAGACCTGGCAAACAACATAGTGGTGAGGATGGTGGCAGGAAAGCGATATTTTGGAACTGGTGCGTATG
GAGATGAGGAGTCGAGGCTGTTCCAAAAGGCCTTGAAAGATTTCATGCATCTGCTGGGGTTGTTTATGGTTTCTGATACAGTTCCCTT
ATTTGGTTGGATAGATGTATTGAGAGGATACAAGGGTAAGATGAAGAAGACAGCTAAGGAGATCGATCATGTACTTGGAAGCTGGGT
GAAGGAACATCGACAGAAAAGGGAAACTCTAAGCATCAATGAACCAGAGCAAGACTTTATTCATGTTATGCTATCAGTCATTGATGGT
AGTCAGTTCAGCGAGCTTGACACTGATAAAGCCATTAAGGGCACTTGCTTGAGTCTTATCTTAGGAGGCTATGACACAACAACGATCA
CACTTACGTGGGCAGTCTCTTTGCTACTTAACAACCGCCACGTATTAAGAAAAGCCCAAGATGAGTTAAACATCCACGTAGGAAGAGA
CCGCCAAGTTATAGAATCAAACGTGAAGAATCTAACTTACTTACAAGCCATTGTCAAGGAAACTTTACGATTATACCCAGCTGCACCAC
TATCAGTGCCACACGAAGCAATTTCAGACTGCACAGTGGCAGGCTTCCATATTCCAGCTGGCACTCGGCTACTAGTTAACCTCTGGA
AGTTGCATCGGGACCCTAGCATTTGGTCCGACCCTCTAGAGTTCCAACCCGATAGGTTCCTCCAGAAGCATGTTGATGTGGACATTT
GGGGTAAGGATTTTGAGCTGATACCATTTGGCTCAGGAAGGAGGTCATGCCCTGGGATCACATTTGCGCTCCAGGTACTGCACTTGA
CACTGGCTCGGTTGCTTCATGGGTTTGAGTTGGGAACTGTCTCAGACTCAACCATTAATATGAATGAAAGTGTAGGAATTACTAACCC
AAAAGCAAGTCCTTTGGAGATTACCCTCGCCCCACGGCTACCTCCTCAGCTTTATGTATAAAACAAAAATATCACCTACAAGTGTGCT

ACTTTCTGCCCTGAAGCTATAAAGCTAGGCTGTATGTCCACGCCAGAGCAGCAATAATTGTTGTCTTTCTTTATTACTAAAATGTTATAT
AATGTTCTATGTGATAGTGCATGGATCAGAATTCATGTAAGAACAGTTTGTTTTCATATTAATAAAGAGCAGCACTGTAACCAA

>CYP-9
CCTCCTCCTCTCTTCTTCCTCCCTTGTTTCTCTCTTCACTCGCATTGTCCTAATTAACTGCCAAGTGTCAAAACCCACGTTAATTTTGGT
TTTTTTAGGTTATCTTGGTTTGCTGGCCTGCCAAAGGTGTTTTTCCGGCGGTGGTTATATATAATATTCAAAATTAGGGTTTATGTAATT
ACTGCGCTGATTGTTTAGATGGATACATCGACGGTCATGATCGTTTTATCCGTTATGGCGGCTTATTGGTTGTGGTTTAGGGCCATGG
CGCAGTCGCTGAAGGGTCCACGTGTTTGGCCCCTATTGGGCAGTCTTCCGGGATTGATACACCATTCTAACAGGATGCATGACTGGA
TAGCTGACAACTTACGCGCGTGTGGCGGCACGTACCAGACCTGCATATCTGCGATCCCGTTTCTAGCGAGGAAGCAGGGGCTCGTG
ACGGTCACGTGCGATCCTAAGAACTTGGAGCATATTTTGAAGCTTAGGTTCGATAATTACCCCAAGGGCCCTACCTGGCAAAGTGTG
TTCCATGATTTGCTTGGGGAGGGGATCTTTAATTCTGATGGTGACACGTGGCTGTTCCAGCGTAAGACTGCCGCACTGGAATTTACC
ACCAGAACATTGCGCCAAGCCATGGCTAGGTGGGTCAGCCGAGCTATCAAGCTCAGGTTTTGCCCGATTCTTAAGACTGCTCAGATT
GAAGGCAAGCCGGTTGATTTGCAGGACCTCTTGCTTCGGCTCACTTTTGACAACATATGCGGCTTGGCTTTTGGTAAGGACCCAGAG
ACGCTTTCCCCCAGCCTACCTGATAACAGCTTCGCCTTGGCTTTTGACCGAGCTACAGAAGCCACGCTGCAGCGCTTTATTTTGCCC
GAGATGATTTGGAAGCTGAAGAAATGGCTTCGGCTCGGGATGGAGGTCAGCTTGACCCAAAGCATTAAACACGTGGACCAATACTTG
CAATCTATCATCAAGACACGTAAGCTCGAGTTGCTGAATCAGCAAAATGACGGTGGCCGGGTCCCACATGATGACCTCCTCTCAAGG
TTCATGAAGAAAACGGAATCCTACACGGACGAATTCCTCCAACACGTGGCACTCAACTTCATCCTGGCTGGACGTGACACGTCATCG
GTGGCGCTGAGCTGGTTCTTCTGGCTGGTCAGCCGCAACCCAAGAGTGGAACAAAAAATATTAACTGAGATATGCACTGTTCTGATG
GAGACACGTGGCAGTGACACCAACAAGTGGCTTAATGATCCCCTAGTGTTTGAGGAAGTTGACCGATTGATATACCTTAAAGCAGCA
TTATCAGAAACCCTGAGGCTGTACCCATCTGTCCCAGAGGACTCAAAGCACGTGATCTCTGACGATATATTGCCGGACGGCACGGTT
GTTCCGGCAGGTTCATCGATCACATATTCGATATATTCGTCGGGCCGGATGAAATTCATTTGGGGCGAAGATTGCCTAGAATTTCGAC
CAGAAAGATGGTTATCAGAAAATGGTACAAAATTTGAAGCTAAAGATCCGTTTAAATTTGTCTCGTTCAATGCGGGCCCAAGGATATGT
TTGGGTAAGGATTTGGCTTATTTGCAAATGAAGTCAATTGCGGCGGCATTGTTGCTGCGCCACCGGCTGACGGTGGCTGCCGGACA

CCGTGTGGAGCAGAAAATGTCATTGACATTGTTCATGAAATTTGGGCTCAAGGTTGATGTGCACCCTCGGGACTTAACCCCCATATTG
GCTATTATAGGAGAGAAATCTGCATGCATGGGCGAGTCACGTGATGAGGAGCTTGAATATGTTCAACTTGTTGCTGAGGTTGCCTAG
GAATTGGCTGGGGTTTGTGATGAGTCAAATTTTGTTCTCACAATCTCTCTCTCATCTACTGTACTACTTTTATATATTACCGTCCTCACT
TAATCTTTATTTATTTAAATACAATTGCTTGATAGCAAAATAAAGATTACGCGGAGAATAATATATAACAGATAAGATAGAGGATGGAAC
AAAAAAAAAGAAAAATAAATTGAATTCCGTTTGTGATATGTGAATATATCATGTGATGCATATTAGAGAGTATATATATGATCACACATG
TAGAGTTACTGGCTTTGATGATCTTTTGTGACAGGCATATGCAAAACCATTTGATCATGTTATATTGGATTGACTGATCATATAGGATTC
AACAGGATGCATGGCTGTCACAAATGATCATCACTAGCGTCCGTCAAGCTTGAATATTTCCTCAATATACAGCTTCTATGTACTAATAT
TGTTACTTGCTCTTTTTATTTCCCGTGCCAAAAAGGAAGAAAACTTGGC

>CYP-10
CTCTGGTTAAACCCTCTATTGCCACTTCTAAGAAATTTGCCTTCCATTATGGACATAAACTGGTTGTATGATTACTTGCATTTTTCTGACAT
AGCCATTGCATTGTTAGGTCTCTTCTTCATTAGCTCTATACTTGAGAGAGTGGCTAGAAAGGGTCCTATGTTATGGCCAGTACTCGGAAT
GGCTCCTTCTTTAATTCTTCACTTCGACCATATCTACGATTGGATCACGGGATCTTTGATTCAATCTGGCGGAACATTTCACTACAGAGG
AATTTGGAAGGGTGGAGCCTTTGGTATCATGACCGCTGACCCTTCCAAAATAGAATACATGCTCAAGACAAAATTCAACAACTTCCCAA
AAGGGAAATATTATCGGGAAAGGTTCTACGAATTACTTGGGGACGGAATTTTCAATGCTGACGATGATTTGTGGAAGCAACAAAGGCGA
GCCGCAACTTCTGAGATGCACTCAAACCGTTTCCAAGAGTACTCGTTGCAAACCATGCAAGATTTGGTGCACAACAAGCTGTTGAAAC
TCATGGATAATGTTTTGGATAAGTCCACTTGCATTGATCTACAAGAAGTGCTGCTTCGTTTTACATTCGATAATATTTGTATAGCCGCCTTT
GGTGTGGATCCGGGATGCTTGTCACTTGATCTACCCGATGTTCCTTTTGCTAAAGCCTTTGAGGAAGCCACTGAATTAAGTTTGTTCAG
ATTCTTAGTCCCTCCAATTGTATGGAAGACTATGAGGTATCTTGGGCTATGGTCCGAGAAGCGGCTCAAGGAAGCAGTAAGAGTTGTGC
ATGATTTTGCCGAGAAGACGGTGAGACAGAGGAAAGGAGAATTAGGCCACCAACATAACGAGCGGTCCGATCTCTTGTCAAGGCTAAT
CAGTGCTGAAAATGCATATTTCTCAAATAACTTGCTTAAAGATTTTTGCATAAGCTTTATATTGGCAGGGAGAGACACAAGCTCAGTCGG
CTTAGCATGGTTTTTCTGGCTACTGCATAAAAACCCACGCGTGGAAAACAAAATTCTTGTTGAAATTTACGAGATTTTAAAAACAAACAA
AAAAGAAGCAGATTATAATGACGTAGTTTTTACCGCAGAGGAATTAAAGAAGATGGTGTATTTACAAGCAGCATTATCCGAATCTCTAAGA

CTTTATCCTCCCGTGTCTATTGATTTTAAACAGGTTTTAGAGGACGACGTATTCTCGGACGGGACGGCAGTTAAGAGCGGCGCACGAAT
ATTTTACTGCATTTTCTCGATGGCTCGAATGGAGTCGGTTTGGGGGAAGGATTGCCTCGAGTTTAAGCCGGAGAGATGGATTAAAGAA
GGAGAATTTGTGAGCGGGAATCAGTTCAAATATGCGGTGTTTAATGGTGGACCTAGGTTGTGTTTGGGGAAGAAATTTGCATACATGCA
GATGAAAATGGTGGCAGCTTCAATTCTGTTAAGGTATTCAGTAAAGGTGGTTGAAGATCATCAAGTTGTTCCAAAAATGACTACTACGCT
TTACATGAAGAATGGGTTGATGGTAAGTTTGGAGCCTAGGTCAAAGTTGGACCTAGGCCGATATTAATAAAGTTTGCTACGTAACTAGTA
GCAAGCTTTTTCGTTTGGTTGTTTGTTTGTGCAGTAAAAATATTCTCGCAACTTGAGGTCTTGGTTCAATGGCAAAAGCGTGGGAATTC
GAATATCGACAGTGACAAAGTAGTATGTGTGAGTTTAAATTCTGTACTAAATTAACAAAAACAAAAAAAAGTATTAACTCAAGATATCCAAG
AACAAACCTCCTTGTCCACCGGACCAATTCCTTAATAATTTGTGAGTACGTAGTTGTGCAATATTTGAATAAGAAAGAAG

>CYP-11
CGGAAACTCCGCCTGCCGCCGGGAAACCTCGGCCTCCCCTTTATTGGGGAGACGCTTCAGCTCATTTCGGCGTACAAGACTGAGAA
CCCGGAGCCCTTTATTGATGACAGGGTGGCTAAATACGGCACCGTTTTCACTACCCATGTGTTCGGGGAGCCGACCGTTTTCTCGGCT
GACCCGGATACGAACCGGTTTATCTTGCTGAATGAAGGGAGATTGTTTGAGTCGAGCTATCCCGGTTCGATATCTAACTTGCTGGGAAA
GCACTCGTTGCTGCTCATGAGAGGGACTCTTCACAAGAGAATGCATTCTCTCACGATGAGTTTTGCTAATTCTTCGATTATTAAGGACC
ATTTGTTGGTTGACATAGACCGGTTGGTCCGGGTCAATCTGGAGTCTTGGACCGGTCGGGTTCTCCTCATGGAGGAGGCTAAGAAGA
TTACATTTGAGTTGACAGTGAAGCAGCTGATGAGCTTTGATCCATGCGATTGGACACAGAAATTAATGAAAGAGTACATGCTTGTGATC
GAAGGCTTTTTCTGCCTTCCTTTTACTCTCTTCTCCCCTACTTACCGCAGAGCCATCCAAGCTAGAGGAAAGGTGGCCGAAGCGTTGA
GCTTGGTGGTGAGAGCACGGAGGAGGGAGAGCACGGAGGAGGGAGGAGAGAGAAAGAATGATATGCTGGCGGCGCTGTTGGCAG
GAGACGGCGGAGGAGTGGGATTCTCCGACGAGGAGATTGTGGATTTCTTGTTGGCTCTGCTGGTTGCCGGATATGAAACCACCTCTA
CTATTATGACACTGGCCGTTAAGTTTCTCACCGAGACTCCTCTGGCTTTGGCTCAACTCAAGGAAGAGCACGATGAGATTAGAGCAAG
GAAAGGTGAATTTGAGGCACTAGAGTGGGAGGACTACAAGTCCATGCCGTTCACCCTATGTGTTGTTAATGAAACTCTCCGAGTTGCC
AACATAATCGGTGGGGTGTTTAGGCGAGCAATGACAGATGTCAATATAAATGGTTACACGATTCCAAAAGGATGGAAAGTCTTCACTTC
ACTCCGGGCAGTCCATCTAAATCATGAACACTTCAAAGATGCACGAGTTTTCAATCCTTGGAGGTGGCAGAGCAATTCAGGAGCAATAA

AATCGGTTAATGTGTTCACGCCATTCGGAGGAGGACCAAGGTTGTGTCCGGGCTACGAGCTTGCCAGGGTGGAACTCTCTGTCTTCC
TACACCATCTCATCACTCGTCTAAGTTGGGTTCCTGCTGAAGAAGACAAGTTAGTTTTCTTCCCAACGACTCGGACACAGAAAAGGTAT
CCGATAATAGTTCAAAAACGAGGCAAGCCATGTAAAGAGTAAGCAAGTCATCCGAGAAGAATAAAGTTTGTAATCTAGATAAATCAGGC

>CYP-12
GTCACAAAAATACGGTCCCATTATGCAGCTCCGCTTCGGCTCCTTCCCGGTAGTCGTCGGTTCCTCTGTTGAAATGGCCAAAATCTTC
CTCAAAACCATGGACGTAACCTTTGTGGGCCGTCCTAAAACTGCCGCAGGCAAATACACCACATACAATTATTCCGACATGACATGGT
CCCCTTACGGACCCTACTGGCGCCAAGCGCGAAGAATATGCCTAACGGAGCTATTTAGCGCAAAGCGGCTCGAATCCTACGAGTAC
ATAAGGGTGGAAGAAATGAAGGCAATGCTTAAAGAGCTTTACGAGTTGTCGAGGAAGAATACTAGTATTGCCCTGAAAGATTATCTTT
CTACCGTGAGTTTGAATGTGATTAGCCGGATGGTGTTGGGGAAGAGGTATTTGGATGAGTCGGCGGAGAACTCGACCGTGAAGCCC
GAGGAGTTCAAGAAGATGTTGGATGAGTTGTTTCTGCTCAACGGCGTGTTTAATATTGGGGATTCAATTCCGTGGATTGACTTCTTGG
ACTTGCAGGGTTATGTGAAGAGGATGAAAATAGTGAGCAAAAAGTTTGATAGGTTTCTGGAGCATGTGCTTGATGAGCATAACGCCA
GGAGAGAGAAAGAGGGGTTAATTATGTGGCCAAAGATATGGTGGATTTGTTGCTTCAGCTGGCTGATGATCCAACTCTGGAGGTTAA
GCTTGAGAGGCATGGAGTCAAGGCATTTACCCAGGATCTACTTGCTGGTGGGACTGAGAGCTCGGCAGTGACGGTAGAATGGGCAA
TCTCCCAACTTTTAAAGAAGCCTGAATTATTTGAGAAGGCAACCGAGGAACTGGACCGGGTGATTGGAAGGAACAGGTGGGTAGAAG
AGAAGGACATTCAAAATCTTCCTTATGTTCAGGCCATTGCCAAAGAGACAATGAGGCTGCATCCTGTGGCACCGATGCTAGTCCCTC
GGGAAGCTCGAGAAGACTGTAAAGTTGCTGGATATGACATCGTTAAAGGAACCCGAATTCTGGTGAGTGTATGGACCATCGGCAGAG
ACCCTGAATTGTGGGACAAACCAAATGAATTTGTGCCCGAGAGGTTTATTGGGAAGGATATCGATGTTAAGGGCCATGATTTTGAGCT
ATTGCCGTTTGGAGCAGGAAGAAGAATGTGCCCCGGCTATAGTTTGGGATTAAAGGTAATCGAATCCAGTTTGGCCAATCTTTTGCAT
GGATTCAACTGGAAGTTGCCTGATTCAATCACAACTCAAGATTTGAATATGGACGAAATATTTGGGCTTTCCACTCCTAAAAAGATCCC
ACTTGTTACGGTTGCTCAGCCTCGACTACCACTGGAGCTTTATTCTCTTTAGAACTACAACTAATAAGTTTGTCATGTTACTATATGCT
GTAGTTTCTTAATTCACCAAAAGTAAGCTT

>CYP-13

CTTTCATCCTCAGAGTTAAAGACAACAAATACTCGGGATTTTTATCCTTATTCTCCACTTTTATTTTGAACCTCTCAATTACTGACGCAGC
AATTGACTTCATTTGAATATAGGCCATATCTTTTCCTAGGCAAATCCTCGGCCCCGCATGAAATATCGGAAACTTAAATGCACTCTCTTGC
TTACACATGCCGTTTTCGTCAAGCCACCGTTCCGGCCGATATTCACAACAATCTTCCCCCCAGATATTCTCCATTCTAGCCATCGCATAA
GCATTATACGTCGCGAACCACCCCTTTTTAATAATCATACCGTCCGGCCAAATGTCGTCGTTTAGGCACGCTTTAGTATCAACCGACACG
GGCGGGTACAATCGTAACGCTTCCGATATTGCTCCGTGGAGATATTGCATTTCACGTAACTCGTCAAAACTAAACGTATCCCCTCTATTT
TTACGGCCATTTTCCCGGATTAATTTGAGCTCTTGTAGTATTTTTTCAGTTACATCCGGTCTTGAAGATAATAGCCAAAAAACCAAGTCAA
AGACGATGAGGTCGTGTCTCGGCCGGCTAAAATGAAACTAATGACGATATCTCTGAGGTATTCGGCCGAGTTTTTGGTTTCCGTCATGA
ACCGGGATAACAAGTCTTGGTCTTTCCTTTCTGCCCGCTCTTGCAATCTTGACCGTATGATCTTATCCGCAAACTCATGGACGATCTTG
ATTGAATTCCGTAGTTTTCGCTCCGTTCCGATATTGAGGAATTTTTTAATATCGCAAAACACCGGTAAGATATACATAAATCGGCCGGCAC
TTAGTGTTGCGGCTTCTTCAAACGCTTGCATGAACTCGCTCCCGGCGGTTCCATCGCCTTTCAGGCAACCCGGATCAACATTAAAAGC
CAACTTACAGATATTATTGAATGCAAACCGCTCTAAAAGGTCTTGGATATCCAAAA

>CYP-14

GAGCGTAGAGAAGCTGCTGGAAGAGAAACGCCGGGCCGAGTTGTCTGCTCGTATTGCTTCAGGAGAATTCACTGTAGAAAAATCTGG
TTTTCAATCACAGTTGAAGAATAGTTTGTCCAAATTGGGGTGCCTAGTGATATCCTTAATTTCTTATCTAAATGGATTGATGCTGGTGACA
ATTATCCAAAGATTCCAGAGGCAAAAGGAGCAATTAGTGCTATTCGAAGCGAGGCATTTTTCATCCCCTGTATGAGCTTTACCTCACTTA
TGGTGGAATTTTCAGGTTAACATTTGGTCCCAAGTCCTTCTTGATAGTTTCTGATCCTTCCATTGCCAAACACATATTGAAGGATAACTCA
AAGGGTTATTCAAAAGGTATCTTGGCAGAAATTTTAGATTTCGTAATGGGTAAGGGACTTATCCCAGCTGATGGGGAAATATGGCGTGTT
AGACGACGTGCTATAGTTCCATCATTGCATCAGAAGTATGTAGCAGCGATGATAAGCTTGTTTGGACAAGCTACTGATAGGTTGTGTAGA
AAGCTTGATGCTGCTGCATGTGATGGGGAGGACGTGGAGATGGAGTCACTTTTCTCACGTCTAACATTGGATATTATCGGGAAAGCAG
TCTTTAACTATGATTTTGACTCTTTAACTAATGACACTGGGATAGTTGAGGCTGTGTACACTGTCTTGCGAGAAGCAGAAGATCGAAGTA
TCGCACCAATCCCAGTTTGGGAGATTCCTATTTGGAAAGATATTTCACCAAAGCTAAGAAAGGTCAATTCAGCGCTCAAGCTGATTAATG

GTACACTGGACGATCTCATTGGCATATGTAAGAGGATGGTAGAAGAAGAAGAGTTACAGTTTCATGAGGAGTACATGAATGAAAAGGAT
CCTAGTATTCTTCACTTTCTATTGGCATCTGGAGATGATGTCTCAAGCAAGCAACTCCGTGATGATCTGATGACAATGCTTATAGCTGGA
CATGAAACAACTGCTGCACTGTTGACATGGACCTTTTATCTTCTTTCCAAGGAACCTAGTGTCTTGTTGAAGCTTCAAAATGAGGTCAAT
TCAGTTTTAGGTGACAGGATTCCAACCATTGAAGACATGAAGAAACTCAAGTATACAACTCGAGTAATCAATGAATCATTGAGGCTCTAC
CCACAGCCACCTGTTCTGATTCGACGTTCTCTTGAAGATGACAAACTTGGGGAGTACCCAATAAGAAGGGGTGAAGATATCTTTATTTC
TGTCTGGAACCTACATCGCTGTCCTAAAAGGTGGGAAGATGCAGATAAGTTTAATCCTGAAAGATGGCCCTTAGATGGACCCAACCCAA
ATGAGACCAACCAATCTTTTAGTTATTTACCATTTGGTGGAGGACCAAGGAAATGTGTAGGAGACATGTTTGCATCATTTGAGGCTGTAG
TAGCAGTTGCAATGCTTGTTCGGCGATTCAACTTTCAAATGGCACTTGGGGCACCTCCGGTCAAAATGACTACGGGAGCAACAATCCA
CACAACAGAAGGATTAAATATGACAGTCACAAGAAGGATAGAACCTCCAGCTCTTCCTACACTTGAGATGCAGATGTTGAAAGTTGATT
CATCCGTTAACATGTCTGAAGCGGACCCAGTGGTTGGTCAAAAAGGTGAAGTTTCTCCAGCTCATTCCTAGTATTTCAACAGCCTTGCC
ACTTTGTAGCAACAAAGCATTAGATATTATTAACACTGAGGAAGGTAGTGAGAGCAATTAGTTTATCAAGTTCTTCATTATCTTGAACCTAC
TCTCATTCTTAGCGG

>CYP-15
GCCTTACTTCTATTTGCCTTCTCTCTCTTAATCTTATAATCTTTCTCCAAAAACTCTATCAACGGCTGAGATTCAAGCTCCCACCGGGGCC
CGACCGTTGCCGATAGTCGGTAACCTTTACGACATCAAGCCCCTCAAGTTCCGGTGCTTTGCCGAATGGGCCCAAGTCTATGGCCCTA
TCTTTTCCTTGCGTCTGGATTCGCGGTTGAACGTGGTCGTTACAACGCGGATTTGGCGAAAGAGGTGCTTAAAGAAAATGACCAGTAT
TTGGCAGATAGGCATAGAAATAAAGCAACTGATATATTTAGTAGGGGTGGCCAAGACCTGATATGGGCTGATTATGGACCTCACTATGTT
AAGGTTAGAAAAGTATGCAATCTTGAGCTCTTCACTCCAAAGAGGCTTGAAGCTCTTAGGCCAGTTAGAGAAGATGAAGTTACAGCCAT
GGTTGAGTCCATTTTCAAGGACTGCACCACCACTTCAGACAAGTCGGGGAAGAGTTTGGTGTTGCGGCCTTACTTGGGGTCAGTAGC
ATTCAACAACATAACGAGGCTCTCATTTGGAAAACGATTTGTGACCACCCAGCGGGGAATGGACGAGCAAGGCCAGGAATTCAAGGG
AATTGTGTCAAATGGTATAAAGATCGGAGCCAAAGTTTTCATGGGAGAATACGTGCCATGGCTACGTTGGATGTTTGCAGGAGAAAACG
ATGTACTTAATCAGCACGAAACGCGTAGGGCTCGGCTCACCAGGCAGATCATGGAGGAACATACCCTTGCCCGGAAGAAAACCGGTG

GCGCAAAGGATCATTTTGTTGATGCATTGCTCACTCTTCAGAAGCAGTATGACCTTAGCGATGACACTGTTATCACACTTCTCTGGGAC
ATGATTACTGCCGGCATGGACACAACTTCAATCTCAGTAGAATGGGGTATGGCCGAGCTAGTTAAGAACCCAAGGGTCCAACGAAAGG
CTCAGGAGGAGCTGGACCGCGTGATCGGATCCGATCGGGTCATGACCGAATCCGACTTCTCCAAGCTCCCTTACCTGCAATGTGTAG
TCAAGGAAGCCTTAAGATTGCACCCACCTACCCCTATGATGCTTCCTCATAAAGCCAGTGTCAATACCAAACTCGGTGGCTATGACATC
CCTAAAGGATCCATCGTGCATGTTAACGTGTGGGCGATTGCCCGTGATCCTGCAGTGTGGAAAAACCCTCTCGAGTTTTGGCCTGAGA
GGTTCCTTGAGCAGGATGTTGACATGAAAGGTCATGATTTTCGGCTACTGCCATTTGGAGCCGGAAGGCGTGTCTGCCCAGGCGCTC
AGCTTGCCATTAACTTGGTGACATCGATGTTGGGACATCTTTTGCACCATTTTTCCTGGACACCGCAGGAAGGGGTCAAGCCTGAAGA
AGTAGACACGACAGAGAACCCTGGGATGGTCACTTACATGCGAACCCCGTTGCAAGCTATTGCTACTCCAAGATTGCCCGCAGAGTTA
TATAAGCGTGTGCCTGTGGACATGTAATCCCCAATTTTTATTATTCTCCCAATATGTTGTGTTTTTATAAATTCTTCGTGAGATGCATTGTTT
TATATTTGATTATGGACGGG

>CYP-16
GATCTATCCCATAAGATCTTTTCCAATGTCCGTCCCGATGCCTTTCGTTTGGTGGGTCATCCTTTTGGGAAAAAGCTCTTTGGCGAGCA
CAACCTTATCTACATGATGGGTCAGGATCATAAAGATCTCCGCCGCCGCATCGCCCCCAATTTTACGCCGAGAGCCCTCTCGACCTACA
CAAATTTGCAGCAATCAATCATTCTCAAGCACCTCGCCTCCTGGTTGGATGTGGCTAAGTCTAAGTCGCCGGAATCCATCCCTCTCCG
GATTCTCTCCCGTGACATGAATCTGGAAACCTCACAGAAGGTGTTTGTGGGTCCGTATCTCACATCGGAATCTCGTGAAAGATTCAATT
ATGATTACAATTTTTTTAATGTGGGATTGATGAAGCTGCCGATAGATCTGCCTGGTTTTGCATTTAGAAATGCTAGACTTGCCGTGTCCA
GGCTTGTTGAAACCCTGGGTGGTTGTGCGGAAGAGAGCAAGGTCAAGATGAAGGCCGGAGAGGAACCGACCTGTTTGATTGATTTCT
GGATGCAGGATACTCTCAGGGAGATCGCCGCCGCCGAAGATGCCGGAGAGCCTACGCCGCCGCATTCCGGAAGTATTGAAATCGGT
GGTCATCTGTTTGACTTCCTTTTCGCCTCCCAGGACGCCTCCACGTCGTCGTTGCTGTGGGCTGTGACGCTGCTGGATTCCCACCCC
GGAGGTTCTGGACAGAGTTCGACGGGAAGTGGCCGGGATTTGGTCACCGGAGTCAGACAAGCTGATCACGGCGGAACAGCTGAGG
GAAATGAAGTACACGGAGGCGGTAGCAAAGGAGGTGATCAGAATCCGGGCGCCGGCAACCCTGGTGCCCCACATTGCCGCCGAGG
ACTTCATGTTGACTGCCGACTACACGATTCCCAAGGGAGCTATTGTTTTCCCATCCGTCTACGAGTCTTCGTTTCAAGGCTTCACTGAA

CCGGACCGGTTCGACCCGGACCGGTTTATGCCAGAGAGGCAAGAGGATCAGGTTTACAAAAAGAACTTTCTGGCGTTTGGTGCTGG
GGCCCACCAGTGTGTGGGCCAGAGGTACGCCATCAATCACCTGGTCTTATTCATCGCCATGTTCACAACCTTGGTTGATTTCAAGAGG
CACAGAACGGACGGCAGTGATGATATCTATTACGTCCCAACCATTTGTCCCAAGGACGATTGCAGAGTTTTTCTCTCCAAAAGGTGCAC
TAGATTTCCGTCCATCTGATAACGAAGTTACCCCTTGAGTAGAATTCTCTTCTCGTTGGTAGAGATTGTCATTATCTTCATTTGTTAGGCG
TGTTTCGGTCAGGTAA

>CYP-17
ATTTTTCTCATCAAATTTTCATTACAAAACACAGAAATACTGATTCAAAAATTCCCCTCCCGCCAGGTCCAACAGGCTGGCCTTTGATA
GGCGAAAGTTTAAATTATTTGTCTACTGTCAAAAGTGGGCTTCTTGAAAATTTTGTGACATATAGGAAGGAAAAGTACTCCACCAAAGT
TTTCAGGACATCACTTTTTGGAGAATCAGTGGCAATCTTGTGTGGCGCTGAGGGGAACAAATTCCTCTTCTCCAATGAAAGAAAGCTA
GTCCGAGTTTGGTTCCCGAGGTCTGTCGAAAAGATCTTCGCCCAATCTCATGCCGAATCCAACGCAGAGAGCTTCTACAAAATACGC
AAAATGATGTTTATTCTCAAGGCGGATGCACTGAAAAAATATGTTGGGTTAATGGACACGATCATGAAACAGTTTTTACAGACCCACTG
GAATCATCATCTCCAAACACAGATCAATGTCCATAACACGGTTATGAATTACAGCCTCATGTTGTCGTGTCGGGTGTTTATGAGTATTG
ATGATGCAGAGCAAGTTAGAAAAATCGGCAACTCAATTCACCACATAGAGGCTGGACTCTTTGCTGTGCCTATAAATTTACCAGGGAC
CGCTATGAATCGTGCCATAAAGACAGTGAAGCTGCTATCTAAAGAGTTTGAGGCGGTGGTTAAGCAAAGAAAAGCGGATCTTTTAGA
GAATAAGCAAGCGCCCCCAACGCAAGATTTGTTGTCACACTTGCTACTTACGCCCAATGAGGATGGCCGGTTTATGAGCGAATCGGA
TATTGCTAGGCAGTTGTTAGGATTGGTGCAGGGTGCCTATAGCACATTAAATGTTGTAATCGCCTTCATTATTAACTATCTTGCAGAGC
TTCCTGATGTCTACGATCAAGTCCTTAAAGAGCAAGTGGAAATAGCAAAGTCAAAAAATCCAAAAGAGTTGCTTAATTGGGAGGATTT
GAGTAAGATGAAGTATTCGTGGAATGTTGTTCAAGAGGTATTGAGAATAAGATCACCAGCAATTGGAGTATTCAGAGAGGCTATTAAC
GACTTCACCTATGCTGGATATTTAATTCCAAAGGGATGGAAGCTACATCTGATTCCAGTTGCCACACACAAGAACCCAACATATTTTCC
AAATCCAGAAAAATTCGATCCAACGAGGTTTGAAGGAAGCGGTCCGGCTCCATATACCTTTACTCCTTTCGGTGGGGGCCTCGAATG
TGTCCAGGAGTTGAATATGCACGTCTAGCAATACTCATTTTTATGCACCATGCGGTGACAAACTTCAGGTGGGAGAAGCTCATTCCTA

ATGAACAAATTTTCACTTTTCCAGTCCTAAGTTTTGCGAATGGTCTTCCAATTCATCTGCATCCCCACAACCCCTAAATATATTTGACTA
CTACTCTTATATATATATATATATATAATTAAGCTTTCATCTTTTTGTGTTTTGTAATCCTAATGTATTGTTGCGATGTTGTTTC

>CYP-18

ATTTGCATTAGTCCCCTTTCTAGCTAACTAGAAAGCAAAAAAAGGCTATCAAAAAATGCAACTCTTCTATGTCCCTCTCCTCTCCCTCTT
TGTGCTCTTGGTCTCTTTATCATTCTACTTCCTCTTCTACAAGTCCAAATCCGGCTCGCCTGGCGCGCTGCCTCTCCCGCCAGGCAAG
ACCGGGTGGCCGGTTATCGGAGAGAGTTTCGAGTTTCTCTCCACCGGGTGGAAAGGCCATCCGGAGAAGTTCATATTTGACCGATT
GGCCAAATACTCCTCCAACGTCTTTAGGACATCTTTGTTGGGACAGCCCGCCGCAGTTTTCTGCGGCGCAGCTTGTAACAAGTTCTT
GTTCTCGAACGAGAACAAACTTGTTCAGGCCTGGTGGCCCGACTCCGTAAACAAAGTTTTTCCCTCCTCAACCCAAACCTCTTCCAAA
GAAGAGGCCATTAAGATGCGAAAGTTGCTGCCAAACTTCCTCAAACCGGAAGCCTTGCAGCGCTACGTGGGAATCATGGACCAAATT
GCCAAAAAACACTTTGAATCCGGTTGGGAAAATAAAAAAGAAGTAAGTGTATTTCCCCTAGCAAAAAACTACACCTTTTGGATCGCGT
GTAAGGTTTTTCTCAGCGTAGAAGAACCCACGCAGGTTGCCAAGCTTTTGGAACCTTTCAACGCCATTGCGTCGGGTATCATATCTGT
CCCAATAGATTTGCCCGGTACGCCGTTTAATAGCGCCATAAAATCATCAAAAATAATTAGGGACAAACTTTTGGGGATTATAAAGCAAA
GGAAAATTGATTTAGGGGAGGGAAAGGCTTCGCCCACACAAGATATATTGTCGCACATGCTTTTGACAAGTGATGAAAATGGCAAGTT
TATGACCGAGGGGGATATTGCTGATAAGATACTGGGGTTGTTGATTGGAGGGCATGACACGGCAAGCTCTGCATGTACTTTTGTTGT
CAAGTTTCTTGCGGAGTTGCCCGAGATTTATGAGGGAGTCTACAAAGAGCAAATGGAGATAGCGAAATCGAAAAAGCCAGGGGAACT
GTTGAGTTGGGAGGACATACAAAAGATGAAATATTCATGGAATGTAGCCTGTGAAGTCCTCAGACTTGCACCACCTCTCCAAGGTGCT
TTTAGAGAAGTCCTCACTGACTTCAGCTACAACGGTTTCTCCATCCCTAAAGGCTGGAAGTTGTATTGGAGTGCAAATTCAACCCACA
GAAACTCAGAGGTGTTCCCGGAGCCACTAAAATTTGATCCATCAAGATTCGAGGGAGCCGGGCCGCCGCCGTACTCGTTCGTGCCG
TTCGGCGGCGGACCGAGAATGTGCCCCGGGAAAGAGTATGCCCGGCTGGAAATACTGGTGTTTATGTACCACGTTGTGAAGAGGTT
CAAATGGGAAAAGGTTATCCCTGATGAGAAAATTGTTGTTAATCCCATGCCTATTCCTGCCAGCGGACTTCCTGTTCGCCTGTTCCCT
CACAAAGCCTAAGTAAGCTTCAGCCTTCGGGTGGTGGTGCTTTAATTTCGATACTCCAATGGTTAATTGCTTGTATTAATTTTCGTTAA

TTAATTTACTTACTTTTTATTTTAAAAAAAAAAAAAAAAGAAAAAAAAAAAAGAAAAAAAAAAAATAAAAAAAAAAAACAAAAAAAAAAAAGAA
AAAAAAAATAAAAAAAAAAAAATGAAAAAAAAAGAAAAAAAAAATAAAAAAAA

>CYP-19
CCTCCTCCTCTACTACTTCATTCTTCATTCCCCTTCTTATAATTATACTACTAGTAATCCTTCATCTCATCATCAAAAAAAGCAAAACCAC
CAAAAACATCGCCAATCTCCCTCCGGGAAGTTACGGCTGGCCGGTCCTCGGCGAGACCCTCTCATTTCTCCGGGCCGGATGGGACG
GAACGCCAGAGAGATTTGTGATGGACAGGGTGGTGAAATACGGGGTGCTCCGGTGTTCAAGACGTCGATGCTAGGCGACTCGATGG
TGGTTTTTTATGGGACTGCGGCGAATAAGTTTTTGTTTCAGAATGAAGATAAGCTGGTGGCTTCTTGGTGGCCTAGATCGGTGAAGAA
GCTGTTTGGGACTTGTCTTGTTACTGTGGCCGGTGAAGAGGCAAAGTGGGTGAGGAAAATGCTGTATCCTTATCTCTCTCCTGATGC
CTTTGATCGCCTCTATATTAATTCCATGGATACCATTACGCAGAAACATATCACCCACTACTGGCAAGGTAAACAGGAGGTAAAAGTCT
ACCATACCGTCAAGTTATACGCCTTTGAGCTTGCTTGTCGCCTATTCATGAGCCTCCAACACCCAAAACAAATCGAAAACTTAGCCTC
CCAATTCAACATATTCTTAAAAGGGGTCATACAAATCCCTCTAAACATCCCCGGTACAAGATTTTACAGAGCAATGAGAGCTGTAACTG
CCATTAAAAAAGAGCTTTTAACATTAACCAAACAGAGGAAATTAGCCTTGGAACAGAAGACAGCTTCCCCTCTCAAGACCTATTGTCTA
AGCTGCTAGTCTCTTGCGATGAAAATGGAAGGTTTCTGAACGAATCTGAGATTGTTAATAATATAGTGACACTACTAGTTGCTGGCCA
CGACACTTCAACCATTTCCATAACTTTACTCATGAAGACTCTCGCGGAACTGCCTCAAGTTTATGAAAAGGTTTATCAAGAGAATATGG
ATATAGCAGCATCTAAAGGCCCAGGGGAGTTACTACAATGGGATGACATACAGAAGATGAAGTACTCATGGAATGTTGTCCGCGAAG
TTATGAGAATATCACCACCTGTATTCGGTGGTTTTAAAGAGGTCTTGGTGGATTTTACCTATGCAGGTTACACCATTCCTAAAGGGTG
GAAGTTATACTGGTGTACTGGTTCAACACAAAAGGATGAGAAGTTGTTTCCAAATCCCACAAATTTTGATGCTTCGCGGTTTGAAGGA
GCAGGGCCTGCTCCATATTCTTATGTTCCTTTTGGAGGGGGGCCTCGGATGTGTTTAGGGAAAGAGTTTGCTCGGCTGCAAATTATG
GTATTTCTGCACAATATGGTGAAGAGGTTTAAGTGGGGTTTAGTAATTCCTGATGAGAAATTTGAATATGATCCCATGCCTACCCCAGT
AAAAGGACTTCCTGTGCATCTTCAACCTCACACAGTCTGAACTTAACTAGTTAAGTACAAAATAAAATGCTGCACTTTAGTAGACACAC
TGCATGATCAAGTTAATTTTAGCAGTAGTGAGGTCTAAATAAGAGAGCACTAGTAGCCTTCTACATGAGATGAAAATGAAGTGATTTAA

TCACTGTTATCGCATATGTGTTATTATGTGTTATTATGTCTTAAACTACCTATATAATGGAAAAGGAGAGAGTAAATACTCCAACCAATT
AGCACTTT

>CYP-20
GATAAAGGGATTGGAAAAGGAGGTGGAATCATTGATATGGGAAGCAGTAAAAGAACGCGAAAGAGAATGTCTAGAATCGTCGTTATCTG
AGAAGGATCTATTGCAGATGATACTCGAGGAGGCCATCAATGATGTTTATGTTGGCAAAGAGTCATCCAAGCGGTTCATTGTTGATAATT
GCAAGAACATATATTTTGCTGGCCACGAGTCCACTGCCACTGCCGTGTCATGGTGCTTAATGCTTCTTGCTTTACATCCAGAGTGGCAA
GGTCGTATACGAGAGGAGACTTTAGAAATTTGCCAGAATTGCATGCTAGATTCAGATTCGCTCCCCCAAAATGAAAACGGTAACTATGG
TAATTCAAGAAGTGCTGCGCTTATATCCACCAGCAGCATTTGTGTCTAGGGAAGCACTGGAAGAGACTCAGATTGGACGTATAACTGTC
CCGAAAGGCGTTTGCATATGGACACTGATCCCTACGCTGCATCGCGATCCAGATATTTGGGGAGAAGACGCGAATGAGTTCAAGCCGG
AGAGGTTTAGTAATGGAGTGTCAAATGCATGCAAAGTTCCCCAGGCCTACGTTCCATTTGGACTGGGTCCTAGGCTGTGCTTAGGCAA
AAACTTTGCCATGGTTCAACTCAAAGTTGTCATCTCAGTTATTATCTCCAACTTCACATTTTCATTGTCTCCAAGTTATCAGCACTCTCCT
GCATATAGAATGATTGTAGAACCTGGGAATGGTGTATCCATCCGCATTCAAAGATCTAAACAAGGTTGGCAGTAACTGTTTGGAAAATTG
TACAAATTCAGCCCCAAAATTTCTCTTTTTGTCGAAACTGAATTATACTACAATTTTAACAT

>CYP-21
GTACTACAAAGATCGACTCGTTGGCCGGGATGAGAAGGCAGGAGCTTGGTGGGTTGGTGGAGTCATTGATGGAGGCGGCGGTGGC
GCGTGAGGTGGTGGATCTTAGTGAGGAGGTGGGAGGGTTGGTTGAGGATATGACATTTAGGATGCTTTTTGGGCGGAGTAAGGATG
AGAGATTTGATCTTAAGGGTAATATTAAAGAGTTAACTACTGTGCTGGGAGCTTTTAATGTAGCTGATTATGTGCCATTTCTAGGGGTA
TTTGATCTACAGGGTTTGACTCGACGTATGAAGGCAAACTACAAGGCTCTTGACAAAATCTTGGAGATTATAATTGATGAGCATGAAC
AAGATATGGTAGTGACGAGCATAAAAAAGCACCATAGGGATTTTCTTGATGAATTGCTATCATTGAAGAACAACCCTACAAGGACACA
TGAGGAACTGGCTAATAAAATTGACAAAACAAATATCAAAGCCATCATACTAGACATGATCACCGGATCAATTGATACCTCGCGTACC
GGAATCGAATGGATTTTGTCGGAACTTATAAGGCATCCAAATGTAATGAGAAAGCTTCAAGAAGAAATCAAGATTATTGTTGGAGATAC

TAAAATAGTAGAGGAAATAGATTTGCCCAAGTTAAAATATCTAGATATGGTAGTGAAGGAGAGTTTGAGGATACATCCAGTTGCACCA
CTATTCGTTCCGCACGAGTCTATGGAGGATATTGTGATCAATGGATATTACATACCAAAGAAGTCGCGGATTATCTTCAACGCTTGGG
CATTAGGACGAGATCCTAATGTGTGGTCTGAAAATGCTGAAGAATTCTTTCCCGAGAGATTTATTGGGAACAATATAGATCTTCGGGG
TCGCGATTTTCAACTTTTACCATTTGGTTCTGGTCGAAGAGGATGTCCCGGGATGCATTTGGGATTGATTAATATTCGTCTAGTGGTG
GCTCAATTGGTGCATTGTTTCAACTTTGCTCTACCAAATGATATTTCACCCACTAAGTTGGACATGGATGAAAAGTTTGGTCTAACAGT
GAAAAGAGCAAAACACCTGCTTGTAGTACCAACTCTTCGCCAGTAAAAAAGGTTGTATATACTGATCTAAGCGTGCTTCTTATATCTTT
GTATAATTATAATCCATTAATAAGATGTACTTCAACATATTAGAAACTCACCACTGCTTTTTTTTAAAAATTAATAAAAAAATATCACTTA
ATTTCCTCTTTTTTACTTCTAAATTTTTATTTGATTCAAGTAAAAGCCAGCATATATTACTATTATTATTTTGTTCTCTTGTTACCCTGCAA
AATTCAAATTAATTTTCATTTTCTTATCCC

>CYP-22
CTAAATGAAATATGGTGGACTCTAATTCGCTTACAGCGAGTCCTTAGACAACAGGGAATCAAGGGTCCTTGTTACCGATTCCTTTATG
GAAACACGACAGAAATCCTACACATGAGGAAGGAATCCATGAGCAGACCCATGGACTTATCACACAACATATTTCCAAGACTTCAGCC
TCACCTTTACTCTTGGGCTGAACGTTTATGGGAAGAATTTTGTGATTGGCATGGTCCTAGAGCTCAATTTGTCGTTACAGAAGCAGATT
TTGTTAAAGAAACAATTATTAAAGATCAAGCTTATCCAAAAATGGACCCGGAATGGTTTGCAAAGATGTTATTAGGAGACGGCATTGCG
ACATCAAAGGGCGAAAAATGGGCAAAGCATAGGAAACTGGCCAATCATGCTTTCCACGCAGAGAGCTTGAAAAGTATGACTCCAGCA
ATGATTGCAAGTGTCGAGATGATGCTAGAGAGGTGGAAACAACATGAAGGAAAAGAGATTGACGTTTTTCAAGAGTTCAAGATTTTAA
CATCAGAAGTCATTTCCAGGACGGCTTTTGGGAGCAGTTATTTAGAAGGGAAGGATATTTTTGACATGTTAACACAATTAACTATCATA
CTTGCAAGAAATAGTTACAAAGTTCAGCTTCCTGGTATTAGTCTATTTTATAAAAGTAATGATGAAATCGAAGCAGAGAAACTTGATCA
ACGAATATATGACTCTATTTTGAGGATTATGGAGAAAAGAGAGAAGGCAAGTACCATGAGTGGAGAAGTAGGGAGTTTTGGGACTGA
TTTTCTTGGATTACTTATGAAAGCTAAGAATGATGCTGATGAGAAGAACAGAATCACAGCACAAGATGTGATTGACGAATGCAAAACTT
TTTACGTTGCTGGACAGGAAACTACGACAACTTTGCTTGCTTGGGTTA
