# Supplementary material for: Transcriptomic analysis of Siberian ginseng (Eleutherococcus senticosus) to discover genes involved in saponin biosynthesis
Source: BMC Genomics. 2015 Mar 14;16(1):180. doi: 10.1186/s12864-015-1357-z (PMC4369101; doi:10.1186/s12864-015-1357-z)
Supplement: Additional file 4: — List of CYPs gene-specific primer sequences used for qPCR analysis. [file 12864_2015_1357_MOESM4_ESM.pdf]

Additional file 4. List of CYPs gene-specific primer sequences used for qPCR analysis.

Genes	Primers	
EsCYP-01	F: CACTCTTGAGCTTCCAATGGC	R: ACCTTGGCTTGGAGTGTGT
EsCYP-02	F: ACATGCTCCCCATCCCATTTT	R: GACTAGTAAGCGCGCATGT
EsCYP-03	F: ATGGAATGGACCGACAGCAA	R: TCCTGAATAGCGGGTTTGCC
EsCYP-04	F: ATCTCGGACCGGCTATCGTA	R: CAAAGAAGGGGAACGTCGGA
EsCYP-05	F: TGAAGGAGAGGCCGGGAATA	R: AGGTCGAACAAACTCCTGTCT
EsCYP-06	F: CCCGCAAAACAAACGCAAAG	R: TGAGAGTCAGTATCGGGCCA
EsCYP-07	F: GTTTACTGGTGGTACGACGATC	R: CTTCAGTTTCGGTGTTTCTGG
EsCYP-08	F: TTGGAGATTACCCTCGCCCC	R: ATTATTGCTGCTCTGGCGTG
EsCYP-09	F: CCAAAGGTGTTTTTCCGGCG	R: CCAAACACGTGGACCCTTCA
EsCYP-10	F: GGGTTGATGGTAAGTTTGGAGC	R: ACCAAGACCTCAAGTTGCGA
EsCYP-11	F :CATTCGGAGGAGGACCAAGG	R :CATGGCTTGCCTCGTTTTTGA
EsCYP-12	F: AGTCGTCGGTTCCTCTGTTG	R: CAGTAGGGTCCGTAAGGGGA
EsCYP-13	F: CGCTCTTGCAATCTTGACCG	R: TGCAAGCGTTTGAAGAAGCC
EsCYP-14	F: CAGCCTTGCCACTTTGTAGC	R: CCGCTAAGAATGAGAGTAGGTTCA
EsCYP-15	F: GGAAGGGGTCAAGCCTGAAG	R: TTGGGGATTACATGTCCACAG
EsCYP-16	F: ACGTCCCAACCATTTGTCCC	R: ACCTGACCGAAACACGCCTA
EsCYP-17	F: CAACGAGGTTTGAAGGAAGCG	R: TGAAGTTTGTCACCGCATGG
EsCYP-18	F: ATGTACCACGTTGTGAAGAGGT	R: TTAAAGCACCACCACCCGAAG
EsCYP-19	F: TCTCCCTCCGGGAAGTTACG	R: GAGCACCCCGTATTTCACCA
EsCYP-20	F: TTCAGATTCGCTCCCCCAAA	R: GATGCAGCGTAGGGATCAGT
EsCYP-21	F: CACCCACTAAGTTGGACATGGA	R: AAGAAGCACGCTTAGATCAGT
EsCYP-22	F: TTCAAGCTCTCTGCGTGGAA	R: AATGGACCCGGAATGGTTTG
EsBAS	F: GTAGCCCGGGAGAGCTAGA	R: GTAGTGGCGGCCTCAAAAGT
Actin	F: CTCGCATCTCTCAGCACCTT	R: CCACAGCCAACTGAGTTCACA
