# Supplementary material for: Transcriptomic analysis of Siberian ginseng (Eleutherococcus senticosus) to discover genes involved in saponin biosynthesis
Source: BMC Genomics. 2015 Mar 14;16(1):180. doi: 10.1186/s12864-015-1357-z (PMC4369101; doi:10.1186/s12864-015-1357-z)
Supplement: Additional file 5: — Summary of family classification of the annotated UGTs. [file 12864_2015_1357_MOESM5_ESM.pdf]

Additional file 5. Summary of family classification of the annotated UGTs.

UGT family	Subfamily No.	Unique gene No.	454 reads No.	Number	ORF region	Length	Homology to [species name]	Accession No.	E-value
UGT71	1	2	22						
UGT72	1	4	65	UGT-1	Partial	1234	UGT72B10 [Lycium barbarum]	AB360632	1E-170
UGT73	4	14	72	UGT-2	Partial	1030	UGT73B3 [Theobroma cacao]	XM_007042422	2E-100
				UGT-3	Partial	1415	UGT73C1 [Solanum tuberosum]	XM_006354351	4E-159
UGT74	2	11	60	UGT-4	Partial	1225	UGT74E2 [Vitis vinifera]	XM_003632039	2E-141
UGT75	1	2	22	UGT-5	Partial	732	UGT75D1 [Vitisvinifera]	XM_002263939	5E-105
UGT76	1	1	1						
UGT79	1	4	9	UGT-7	Partial	753	UGT79B3 [Glycine max]	XP_003530810	4E-74
UGT83	1	1	1	UGT-8	Partial	509	UGT83A1 [Prunusmume]	XM_008232649	3E-83
UGT84	1	5	21	UGT-9	Partial	938	UGT84B1 [Vitis vinifera]	XP_002285408	8E-48
UGT85	1	39	309	UGT-10	Partial	806	UGT85A1 [Vitis vinifera]	XM_002280434	8E-92
				UGT-11	Partial	1717	UGT85A2 [Vitis vinifera]	XP_002285770	3E-122
				UGT-12	Partial	1398	UGT85A2 [Vitis vinifera]	XM_002262707	6E-149
UGT86	1	10	23	UGT-13	Partial	1016	UGT86A1 [Vitis vinifera]	XP_002276858	3E-102
UGT87	1	5	5						
UGT88	1	2	23						
UGT89	2	2	16	UGT-13	Partial	989	UGT89A2 [Vitis vinifera]	XP_002268383	8E-108
				UGT-14	Partial	914	UGT89B2 [Stevia rebaudiana]	AAR06921	5E-119
UGT90	1	1	24	UGT-15	Partial	1116	UGT90A7 [Hieracium pilosella]	EU561019	1E-161
UGT91	2	2	2						
UGT92	1	4	4						
unclassified		35	190						
Total	23	144	869						
