# Supplementary material for: Transcriptomic analysis of Siberian ginseng (Eleutherococcus senticosus) to discover genes involved in saponin biosynthesis
Source: BMC Genomics. 2015 Mar 14;16(1):180. doi: 10.1186/s12864-015-1357-z (PMC4369101; doi:10.1186/s12864-015-1357-z)
Supplement: Additional file 6: — Lists of sequences of selected 15 UGT genes in E. senticosus. [file 12864_2015_1357_MOESM6_ESM.pdf]

Additional file 6. Lists of sequences of selected 15 UGT genes in *E. senticosus*

>UGT-1

CGTTACCAAAAACCATAAATTATGTCCATCTTGCACCCGTAAGCTTTGAAGACTTGCCTAAGGATTCCAAGATAGAAATATTTATTTTTCT
AGCAGTGACACGGTCTCTTTCATCTCTTCGTGACACTATAAAGTTGTTGGTTGCCAGAACCGCTTTGGTTGCTCTGATATCAGATCCTTT
CGGGACTGATGCCTTTAGCGTTGCTAAAGAGTTCAATATTTCGCCATACTTATTTTTCCCATCAAATGCTATGAGTTTTGCCTTTGCTTAT
ATGTTGCCAAAATTTGACGAGACACTGTCATGTGAGTTTAGGGAATTACCCGATCCGGTGATAATTCCGGGTTGTGTTCCGGTTCATGG
AGGAGATTTATTGGACCCGGTTCAAGACCGAACCAGTGAGGTATATAAGTTGCTTCTTCACCATACAAAGCAGTTTAGTTTAGCTGAAG
GTGTGTTGTTGAATAGCTTTATAGAATTGGAAGAGGGAGCTATTAAAGCTTTACAAGTGAAAGAACCGGGTAAGTTCCCCGTGTATCCG
ATTGGACCACTTATTCAAACCGGTTCAAGTGATGGGGCGGATCCGTCCGAGTGTTTGAAATGGTTGGACAATCAGCCAAGTTGTTCCGT
CTTATTTATCTCATTCGGGAGCGGTGGGACCCTCTCTTATGATCAGCTTATTGAACTAGCCTTGGGATTAGAAATGAGTGGGCAAAAGT
TCTTATGGGTTGCTAGAGCTCCTAATGACAAATCTTCAAATGCTGCTTTTTTTGGTGTCGAAAGTCAAAATGATCCGTTAAGTTTTTTGCC
AGAAGGGTTTTTAGACAGGACTAAAGATCAAGGTCTAGTGGTGTCTTCTTGGGCTCCACAAATTAAAATTCTAGCCCATGGCTCGACAG
GTGGGTTCCTATCGCATTGTGGATGGAACTCAACGGTTGAGAGCGTTGTTCATGGTGTGCCAATGATCGCTTGGCCACTTTATGCAGA
GCAAAAGATGAACGCGATAATGTTAACTGAGGGGCTAAATATAGCCTTAAGGCCAAAACTTAACGAAAATGGTATAGTGGAGCGTGACG
AGATTGGAAGGGCTGTGAAAAGTCTAATGGAGGGAGAAGAAGGGAAGAAGGTTCGTAGCCGAATGGAGGAACTAAAAAAATGCTGCG
ACAAAAGTACTAAGAGAAGATGGATCTTCCACAAAATCGCTATCTGAATTAGTGAACAAATGGAAGAATAAAA

>UGT-2

ATCTTGATACCGCCAGGAAAGTGTTCACCGGAATGCAACTCCTCCGGAACTCCGTTGAAGAGTATACAACTGAGCACCGGCCGGATTG
TCTTGTGTCGGATATGTTCTTCCCATGGACGGCGGACCTCGCCATCAGGCTCGACATTCCTCGGTTAATTTTTCACGCAACGTGCATGT
TCTCTCAGGTACTGAAAGACGCGGTCCGCCGCCCCGACTCGCCTCACCTGACCGCCAAATCCGACTACGACCCGTTTGTCATTACCGG
GCTCCCACACCCCATCACCATGACCCGAGCCGAGCTCCCGGACTACGTCCGGACGCCGAACGGGTACACCAAGATGATGGAAGAGTG
GAAAGAGGCTGAGTTGAAAAGCTACGGTGTACTCGTAAACAATTACTACGAGTTCGACTCGGCCTACACCGATTTTACCAAAAAATCAT

TGGCCCGGCCTACAAAATCATTAATGTGGGCCCGGCGGCGTTGATTCACCGGAGCGGCAACGAGAAGGTGGAGAGAGGGCATAAGA
CGGTGGTGGGCGAGCACGAGTGCCTGAGTTGGCTCAACTCGAAGGAGCCTAACTCGGTGCTTTATGTTTGCTTTGGGAGTGCGTGTAT
ATTCCCTGATGCGCAGCTCATGGAGATAGCGTGTGGCCTCATTGCTGCGGGGCATGATTTCGTGTGGGTGGTGCTTGGGAAGGATGA
TAAGAAGAAGGAAGATGAGGTGAATTGGTTGCCGTTGGATTTTGATGAGAAGATGATGAAGACTAATAAGGGAATGATCGTCAAGGGG
TGGGCCCCACAGGTGTTGATCTTGGACCATCCATCAGTTGGCGGGTTCTTGAGCCACTGTGGTTGGAACTCGGTGATCGAAGCGGTG
AGTGCTGGCGTCCCAATGGCGACGTGGCCTTTATATGCAGAGCATTTCTACAACGAGAAGTTGTTGACTCAGGTGTTGGGTATTGGAG
TGGAGGTGGGCGCAGAGGAGTGGAACCTTTGGGTAGATGCTGGCAAAAAGGTAATCAAGAGAGAG

>UGT-3
TTTTCTAGTTTCTCCGATGGCTTCCCAAATTGAAGGGCTCCACTTCATCTTGATTCCTTTCATGTCCCAAAGCCATATCCTTCCACTCATG
GACTTTGCTAAACTACTCGCCCAGCGTCACCTAACCGTCACCATAATCACTACCCCCCTTAATGCTATCTCAGTCAAATCCGTAATCGAC
CAGGCCCAAAAAACCCACCTCAAAATCGGCCTAGCCACGGTCCCTTTTCCTTCCCAAGAGGCCGGATTGCCCCAAGGATGCGAAAGCA
TGGAGACTCTACCTTCACACGACATGGCGAAAGATTTTTTTTGTTGGATGTAAAATGTTACAAGAACCAATCCAAAAGCTGCTGGCTGAG
TTACAGCCACGGCCCAGCTGCATCATTTCAACGAATTCTCTTCCTTGGACCGCTAAAGTTGCGTCTAATTTGGGGATTCCGAGGTATGC
TTTCCAAACAATTGCTTGTTTCTCTCTCCTGATGTCCCACAATGTCGGGCGCATGACTGTTGTTCAAACTGCTAGTTCGGATACAGAGCC
ATTTGTGGTACAAGGCATGCCTGATAAAATTGTGATAACTAAATCCCAGATTCCCCAAATTGTAACGAAAGCAAGAGAAGAAGATATGAA
AGGTATTATAGGTGAAATGATTGAGGCTGAGCGTTTAACAAGAGGGATGGTAGTGAATAGTTTTGAGGAAATGGAGCCAAAGTATGTGG
AAGCTTTCAAGAATATGGGAAATAAAATTTGGTGCATTGGCCCAGTCTCTTTGTGTAATAACGAAATGTCGGATAAGTTAGAAAGGGGTG
GTAATAAGGCGTCAATAGATCAAAATCTTTGCTTGAAATGGTTAGATTCAATGGAGTCTAATTCGGTTATTTATGCCTGTTTTGGGAGCAT
GGGTAGAATTCCATCGGCTCAAATTATTGAAACTGGTTTGGGATTAGAAGCTTCAAGTCGCCCATTTATTTGGATCATTAGGAAAAGGGA
TCTTTCTGCCAAAGTAGAAAAATGGTTAGAGGATGAAAAATTTGAAGAGAGAGTAAAAGGGAGAGGCCTAATTATCCGAGGATGGGCG
CCCCAAGTTTTGATATTATCCCATCCAACGGTTGGGGGATTTATAACCCATTGTGGGTGGAATTCGACATTGGAGGCAGTGTGTGCAGG
GGTGCCAATGATAACTTGGCCTATGTTTGCCGAGCAATTTTACAACGAGAAACTGGTGGTGAATGTGTTGAAGATCGGTGTGAGAGTTG

GGGTTGAAGTTGCAATGAAAACGGAGGAAGATGAGGATAAGGTGTATGTTAGTAGGGAGCAAGTGAAGGAGGCTATAGAGCAGCTAAT
GGATGAAGAGGAGAAAGGGGAGAGAATAAAAAGAGCTAAAGAGCTTAGTGAGATGGCTAAAAAGGCTACCGCA

>UGT-4
TCTGAGATGGGCTCGATTCAGATCGAGTCCATCTCGGATGGCTACGAGAACGAAAAACCCGAGAGTATAGACGCATTCGTGGAGCGTA
TTGACTACGTCGTTTCGCAGACCTTGCCCGAGATAATTAAAAAACAAGGCAGCTCTGTTAATCTCCTTGTGTATGATGCAATGATGCCAT
GGGTTTTGGACATTGCAAAAGGGCTTGGACTAAGGGCAGCTGCTTTTTTCACTCAGTCTTGCAGTGTTTGTGCAATTTATTACTATATGC
ATGAGGGGAAATTGAAGGTTCCTTTTGAAGGGGAAAATGTTACTCTGCCAGCTATGCCATTGTTGGAGATCAATGAGTTGCCTGATATG
AAATTGCATCAAAATTTATGGAGCATTAATATAAGCCAATTCTCAAATATTGAGGGAGCAGATTGGCTCTTGTTTAATTCTTTTGATAAGC
TGGAAGTTGAGGTAGTGAAGTGGATGGCAACCAAGTGGCCAATCAAGACCATAGGACCAACTATTCCATCAATTTACTTAGACAACCGT
TTGGAGGATGACAAAGACTATGGCATCAGTCTCTTCAAGCCAAACGACGACGTTTGCATGAAATGGTTAGACACAAAGCCAAATAACTC
AGTTGTCTATGTATCTTTTGGCAGTTCAGCCAATCTTGCAGCAGAACAAATGGAAGAACTAGCATTTGGCCTTTTGAGCAGCAACCACAA
CTTCTTATGGGTAGTTAGAATATCCGAAGAGGGTAAAAATTCCCAGCAATTTCGTGTCCAAAATATCGGACAAAGGCTTGATTGTGAACT
GGTGTCCACAGCTCAAAGTTTTGGCACACAGGGCGATAGGGTGTTTTATGACACATTGTGGATGGAACTCAACGCTTGAGGCACTATG
CTCGGGAGTGCCGATGGTGGCAATGCCACAATGGACGGATCAACCAACTAACTCGAAGTTTATATGGAGGTTTGGCGAGCAGGTGTTC
GTGTGAAAGTTGATGAAAGTGGTATCGTTGCACGGGAAGAAATAGGGGTGTGTTTGAAGGAAGTTATGGAGGGGGAGAAAAGGGATG
AGCTTCGAAGGAATGCAGTAAAGTGGAAGGAGTTAGCTAAAGAGGCAGTGGATGAAGGTGGAAGCTCAGATAGGAATATCGAGGACTT
AATCTCTAGGCTTATAAGCACTTAACTTGTTGGGACTTATAAGCTGGTAATAGAAGTTATAATAAGTC

>UGT-5
AGCAAGTAGAGGCCCTAGATTCCGATACAAACCCTAAAGTACTCGTAAACTCTTTCGATGCCTTGGAGCCGGATGCCCTAAAAGCCATCG
ACAAGCTCAAATTAATGGCCATAGGGCCGCTCATTCCATCCGCTTTCTTGGATGGAAAAGATCCCTCAGATACTTCATTTGGTGGTGATCT
TTTTCAAAAATCGAGAAATTATTCAGATTGGCTGAACTCGAAGCCAAAAGAGTCCGTAATTTATATATCATTTGGGAGCATTTTGGAGTTGA

CAAAGCCACAAATGGAGGAAATTGCTCGAGGGTTAATAAAAAGCAAGCGGCCCTTTTTGTGGGTAATAAGGAAAAAGAAGACGAAAAGA
AAAAGAAGAAGATCAAGAATTAAGCGGCACGGAAGAATTGGAACAATTAGGGATGATAGTGCCATGGTGTTCACAACTGGAAGTTTTGTC
CAACGCAGCGTTAGGGTGTTTTGTGACGCATTGCGGGTGGAATTCGACGTTGGAGAGTTTGGTATCGGGCGTTCCGGTGGTGGCGTTT
CCTAAGTGGACGGATCAAGGGACGAATGCAAAGTTGATCGAAGACGTGTGGAGGACAGGAGTGAGGGTGAAGGGGAATGAAGAAGGG
TTTGTTGATGGTGATGAGATTAGTAGGTGTTTGGAAATGGTGATGGGAGGGGAGGAATATAGAAGGAATGCTAAGAAATGGAGGGATTCG
GCAAGGGAAGCTATG

>UGT-6
TTTTATGGAACCTCCGAGTGACTTTCCTCCTAATTCATCAATCAAGCTAAGTCCACATGAAGCTCGAGAACTAGTCAGATTTTTATGGAA
AAGAGTTCGGTCGAGGCATATCATTCCGAGAGCGAATATTAAAATCCAACAGTGACAGTGATGCCATTTGTATCAAAACGTGTCGAGAA
ATAGAAGGGCAATACTGTGAATATCTTGAAAAGCAATATGTAAACAAGCCTCTTCTTCTAGCCGGGCCGGTTATGTCTGAGCCAACAAA
TTCATCACTCGAACAACGATGGGCTGAGTGGCTAGACCGATTCGAACCCAAGACGGTGATTTTCTGTGCATTTGGAAGTGAGTGGGAC
CTTGAAAAGGATCAGTTTCAAGAACTGGTTCAAGGGTTTGAGCTAACAGGTTTACCTTTTCTGGCCGCCCTAAAACCGCCTGTTGGAGC
TGAGAATAGTGGTATCGAATCAGCATTACCGGAAGGGTTTGAAGAGAGAAATAAAGGAAGAGGTGTAGTCTATGGAGGATGGATTCAG
CAACAACTAATTTTGGGACACCCTTCTGTGGGGTGTTTTGTGAGCCACTGTGGTTCAGGGTCTTTGTCAGAGGGTTTAGTGAGTGAATG
CCAATTGGTGCTACTACCACACGTGGGTGATCATGTAATTAATGCAAGATTTATGGGCGGAGACTTAAAAGTGGGAGTGGAGATTGAGA
AAGGAGAAGAAGATGGTTTATTCACTAAAAGAGAGTGTCTGT

>UGT-7
AGACTGGATAATATGCAATTCAGCCTATGAGCTTGAAACAGGATTAGTTTCATTATTCCCAAAAATCTCGCCAATTGGTCCACTTTCCGC
AGGCAATGGGCTTGGAAATCAAGCAGGCAATTTCTGGCCAGAGGACTCTTCCTGCTTGCAATGGCTTGATCAACAGCCAGCCAGCTCA
GTCATCTATGTTGCATTTGGGAGCTTTACAATTTTTGGCCTAAAACAATTTCAAGAATTGGCTCTTGGGCTTGAACTAACCAACAGGCCA
TTCTTATGGGTTGTGCGGCCTGATATGACTGATGCAACAAATGAAGCGTACCCAAAAGGATTCAAAGACCGAATAGGCAGTCGCGGGC

TAATAGTGAGATGGGCACCCCAACAACAGGTTCTGCGCCATCGTTCTGTTGTGTGCTTCTTGAGTCATTGTGGTTGGAATTCTACCATG
GAAGGTGTAAGTAATGGAGTGCCTTTCTTGTGTTGGCCTTTCCTTGCTGATCAGTTCCTTGAC

>UGT-8
AAAGATTATTTGCTCTTATTTTTTTTATTAATTATTGATCTGTAGCTTTAATACTCTCTTGTAATGGCTTCGGAAGGAAAGAAGAAAGAGACGC
ACGTGCTAATGGTGGCATTTGCAGCTCAAGGTCACGTGAACCCCATGCTTAGACTAGGCAAACGCCTAGTTTCTAAAGGCATTAACGTTA
CATTCGCCGTCAACAAGGATTGCCCTGACCATATAGTCAACTCCCCGCCGCTGCCGGAATCCACCTCGAGTTCTACTCCGACGGCCTAA
GCCTCGACGCCGACCGCATGAACGTGGATCATTTCATGGAAAGCATAGGTGAAGAACTGCTACCGTTAGAGGTTCTTGAAGAAATTGAA
GATCAAGGGCTAATTGTGAAGTGGAGTCCTCAAGTTGAGGTATTAGGGCATCCTTCGGTTGGGTGCTTTTTGACTCATTGTGGGTGGAAT
TCACTGATAGAGACAATCACTGCCGGAGTTCCGGTTATTGCTTGTCCGAAATGGACCGATCAGCCAACAAACGCCAAGCTCATCGACAT
GTTACGTGTCGGAGTGAGGCTTAGGCCGGGCCAAGACGGTGCACTTAGTGGTGAGGAACTGGAAAAATGTTTCGAAGAAGTTATGGTC
GGACCATCATCGGAGGAATACAAGAAAACTGCCGCGGAGTTGAAGCGGGAGGCGAGGGAAGCGGTGGCCAGTGGCGGCTCGTCGGA
CCGGAATATTCAGTGGTTTGTTGATGAGATCATTGGTTATTCTTAATTAAGTCATGACATGTTGTTTAGTAGTAGTATGTAATAATTAATGTTAT
TGCGTTTTTAAGTTGTCCATGGGTAGCCAATCAAAATTTGGTCTTGTGGATGGAACCTATTAGCATGCTTGGAATATGTAATAATTTAAGTTT
GTCACTATTGAAGAACATGATGTGAAAATGAAAAC

>UGT-9
GTAAATTGCATGCTCAAGCTAGCAGAGCTTTTCTCTCTTTCTGAACTCCGTGTCACCTTCCTAAACACCCACCACATCCACCGCCGCCT
GCTAAAGCTCGCCCATGTTGAATCCCGGTTTGCTCGATATCCCGAATTTCGTTTTGAAACCATTTCGGATGGCCTTCCCGAAGACCACC
AACGTTCCGGTGATCAGTTCATAGAGGTCATAAATGGAATAGAAGATGTAACAAAGCCACTTTTCAAAGAGATGTTAGCTTCAGGCTGC
CTGAGTTCTAAGTCAGGCAGGCCTGTAACGTGCATAATTGCAGACGGATTCCTCAGTTTTGTTCTTGAAATTGCTAAAGAGGTTGGAATT
CCACTTCTTAATTTTGAAACAATTAGCCCTTGTGCCCTCTGGACTTATTTGTGTATTCCGAAACTTATTGAGGTTGGTGAGCTTCCTTTCA
AAGGAAATGATTTAGATGCAAAGGTAACAAGTATACCTGGAATGGAAAACTTTCTCCGGCGCCGTGATCTGCCTAGCATTTGCCGGAAC

AATGACCTTAACGACCCAATTATGCAGCATGTTCTGAAAGAAGACCAAGAACTCCCACGAGCTCAAGGACTCATACTCAACACATTCGA
AGACTTAGAAGAACCCATTCTCACTCACATAAGAACTATATGTTCAAATCTTTACCCTATAGGCCCAATCCATGCACTCCACAAGTCTATA
CTTTCAGCAAATGAAAGCCCACCACAAGTCAACTCTTCAAATAGCCTATGGGAAGATGATAAAAGCTGCATGACGTGGCTCGACACCC

>UGT-10
AAGTAGTAGCAAATTGCATTAATAATACCAATTCTAAACCTCATGCAGTTTGTGTTCCATTACCATTTTCCACACACATAAACCATTTGCT
AAAACTAGCAAAACACCTCCACTACAAAGGCTTCCACATAACTTTTGCCAACTCAATGTTCTTCCACAAAACCCTAATTTCTTCCAAAGG
CCCTGCCGCCATGGCGGGCTTACCCGACTTCCGTTTCGAGTCCTATAATCTTGACTACACTCCTCAGCCAAACATACCCCTTACCCAAC
AAATGATGGCCCTTCGCCAGGCCGTTCGGAATCAATTTTTGGCCCCATTTTCTGAGCTTCTTGCGAGGCTCAATGACACTTCTTCAAAT
GTTCCCCCGGTAACTTGCATTGTAGCAGATGGGGTGCTGACTTACGCCCTTCTAGCGGCTGAAAAGCTTGGCATTCCCATTGTACTGTC
ATGGAACGTTAGTGCTAATGGCTTCTTGAGCCTTCATAGTTATCGTGATCTTTTGGCCAGATGTTTTGATCCAAGTAAAGATGTGAGCGA
AGAACAGAGAAATAAGGACTTAGACACCCCTGTTGACTTCATTCCCGGACACAAAGGCATTCCTATTCGAGACATCACCAAGTTTATAC
AAGGTGCAGGTCTAGATTTCCTAGTAGACGGTAGTGTTGGAGAAGCCACAAGAACCTCTAAAGCATCAGCACTAATTTTCTACACTTTT
GAAGCCTTGGAAAAAGATGCATTACACTCTCTCTCCTCCATGTTTCCTCGAGTTTTTAGTATCGGCCCTCTTCCGTTACAATGTTATAGA
AACACGGAGGAGAATGCAAAGTTGGTCGGACACGGTATATGGAGAGAAGAATCGGATTCTGTAAAATGGCTCGACTCAAAGGACACTA
ACTCAGTCATTTACGTTAGTTTTGGCACTACAACTACAGTAGCACCGGAACAAGTTGTGGAGATCGCATGGGGATTAGCCAATAGCGGC
TATCCCTTTTTGTGGATCATTACCCGGGGACAAGTCGAAGGAGTTTTACCACCCGAGTTCATTGAAGAAATACTAACGGATCGAGGCAT
GATAATGGGCTGGTGTGAACAAGAGAAAGTGCTGAACCACGCGGCAATTGGAGGGTTTTTTACGCACTGTGGCTGGAATTCGGTGTTG
GAAAGCGTCGCGGCTGGGGTTCCGATGATTTGCTGGCCTTTTATGGGGGAACATTTGACGACGTGTAGGAATTGTTGCGATGACTGGG
GCTTGGGGTTGGAGATTGATAAGGACGTTACTAGGGAAAATGTGGCCGAGCTTCTGAAAGAGTTGATGGAAGGAGAAAGAGGAAGAAT
GTTGAGGAGTAATGCTAAAGAGTGGAAGAAAAAGACTCAGGAGGCCATTGGTTTGGATGGATCGTCATCTATGAATCTGGAGAAATTAG
TGAATCAAGTGTTATTGGGAAATTAATTATTACTTGGCATGAACTCAATTAGTTTGTTTCTTTTGCTTGCTTGCTTGCCTAGAAGTAAAGT
AATTAATAAGGAGTTGCTTGATGGATTGGAATATTTTCTAATATTGTAATACTCCAGTGTTCTTGTGCTGCATGTGTGTTTCTATATGTTTT

TTCTATTAGAGTTGAAGTTCGGATGTTGTTGTCCAAGCTCTTGGCCGAACGAGGCGCTGTCTTGCCCACTCTCTTTCTATTGTATGTAGT
AACTTTTTCTGTGCAAA

>UGT-11

TCGGGCTCTCCTACTTTCCGTTTTGAAACCATCCCTGACGGCCTTCCAACACCGGAAAATCAAGACGCCACGCAGAACATCGTTGAGCT
TTGCTTGTCCACCTCCGAAAATTGCTCTGTTCCGTTCCGAGATCTACTTAATAAGCTCAACAATTCATCCGATGTTCCTCCGGTGTCTTGT
ATTGTCTCTGATGCCATCATGAGTTTTAGCGTTGAGGTTTCTGAAGAACTTGGCATTCCCAATGTTTTCTTCTGGACGGTAAATGCATTCA
CCTTGATGTGCTACTTGCACTACTCTGCCATCCGTGAGCTAGCTTGTGTGCTACATAAAGATGCAGTAAATGAGTACCTAGACTCCGTTAT
TGACTGGATGCCCGGAACTGGGAGTATCCTTATGCGAGTTTCTTCATCTTTGATTTGGTCACCGGACCTGCCGGATAGCTTTGTCCAGTA
TTGCATTCAAGAAGTTTCAAGAACTTATAAAGCTTCTGCTGTAATTCTCAACACATTTGATGAATTGGAGTGTGAAGTTTTGAAGGCGCTTT
CATCCATGCTGAATCGGGTTTACACCATTGGACCCATTCACAACCTTTCCAAATCGGTCATACCCGATAACCATACCAAATCTTTGAGATCA
AATCTGTGGAAAGAAGATTCGGGTTGTATCCAATGGTTAGATTCAAAACAACACGGATCAGTGGTTTACATAAACTTCGGTAGTATCACAG
TCATGAGTCCTCAACACTTGGTTGAGTTTGCATGGGGACTTGCCAATAGCATGCAAAACTTCTTGTGGATAATCCGACCCGACCTTGTCA
TGGGCGACGCGGCTGTTCTTCCACTTGAATTCGAGATGCAAACCAAACATCGAGGTTTCTTAGCAAGTTGGTGTGATCAAGAACAAGTTT
TGAACCACGCATCAATCGGGGGTTCTTAACACATTGCGGATGGAATTCGACGCTCGAAAGCTTGTCGGCGGGAGTGCCAATGATTTGTT
GGCCTTTTTTCGCTGATCAACTGACGAATTGTTATTGCATTTGCAAATTATGGGGTGTTGGAGTGGAAATTGATAGTGATGTGAAGAGAGA
GGGGGTGGAGAGGGTGGTGAAGGAGTTAATGGAGGGAGAGAAAGGGAAGGAAATTAAGAATAGGGTGGTGGAGTGGAAGAGTAAAGC
TGAGAGTGCTACTAGTCCAGACGGGGGACCATCGTACTTGAACTTGGATAAAATGGTAAATGAAGTGATTCTCTCCGGGCGTGCCCATG
AAGAAGTGTTAATGAAGAACAATGGATTCGAGGGTTTGTCAAGATCGATCATATATAGGTTTTGACTGACATATGAATTTGTTAGTGTGTAT
GTACACTACGTACAAGATTCTTAATATGGTTTTCAAGCTCAA

>UGT-12

CCATTTTTCAAGGCATTAGATGAAGTAAAGAGAGCAGATTTTATTATATGTAACACGGTGGAAGAGCTTGAATCTGATACAATTTCGGCTCT

GCAACAAATGCAGCCCATATATGCAATTGGACCCATATTCCCTAAAGTCTTTACCGAGACCAAGGTGGCTACGAACCTGTGGTCCGAATC
AGACTGCACCCAGTGGCTAAACACCAAGCCTCATGGTTCAGTATTATATGTCTCATTTGGTAGCTACGCTCATACTACTAAGCATGACGTAT
TGGAGATAGCCCATGGGCTTGTACTTAGTGAAGTGAATTTTGTCTGGGTGCTTCGGCCGGATATTGTGAGTTCTGATGATACGGATTTTCT
GCCCATTGGATTTGAAGATGATGTTAAAGATCGAGGGTTGATCGTTCCTTGGTGTCATCAAATTTCGGTGATTTCACATTTGGCGATCGGA
GGGTTCTTAACGCATTGCGGATGGAATTCAGTATTAGAAAGTATATGGTGTTCTGTGCCGTTGATTTGTTTTCCTCTGTTGACGGATCAGT
TCACTAATAGAAAATTAGTGGTGGATGATTGGAAGATCGGAATTAATCTTTGTGACACCTGCAAACCAATTACAAGGAAGGAAGTGGCGCA
AAGTATCAACCGTCTGATGAGTGGAGAAACGTCAAATGAGCTGAGAAATGAGATGAAGACGCTGACAAATGTGTTGACCATTGGTGAGT
CCTCGGAGAAAAATTTCAAAAAGTTTATCAATGACGTGAAAATCAAAATTCTTGAAATTGATAACGGTAGGGGTTCCAAATAATTATTTATTT
TACTCATGCTTAGTGAAAATTAATTGTAAGCAAGATTTGTACTTTTTGTTGTTTTCAAACTTCAAAGTATGAGAAAAAGTGTGGGGACTGAT
CGATGATGGGCTCATGTGCTAAAATAACATATCATGATTATGGCTAACATCCAATATATACGGGTGGTGCTTGTGTCGTGCAATGTTATACAT
CACACAAAAT

>UGT-13
GGTTCAACTCCCATCCTTCTCCCCCTGTTGCCCTACTTTCTGATTTCTTCCTCGGGTGGACCCACCGCCTGGCCCACCAAATTGGAATC
CCGAGAATTTGCTTTTACTCGACGGGTGCTTTTTTAACCTCTATTCTTGCGCGTGCGTGGGATGATGATGTTGACACTCTTCTTTCTTTG
GACGCGGTTGATTTTCCTGATTTACCGCATTCACCCAGTTTTCTTACGGAACATCTCCCTACCGTGTTCCGCGCCTACAGGAAATCAGA
TCCAATGTGGGAGTTCGTTAAGGATGGCATGATTGCAAATAGTGCGAGTTGGGGGTGTGTTTTCAATACCTTTGATGCCGTAGAGGGT
GAGTATTTGGAATACTCGAGAAAGAAAATGGGGCACGAGCGCGTTTTTGCGGTTGGCCCGCTTAGTCTGTTGGGTGTGCCGGACCATA
CGAGTCGGGGCAGCACTATTTCTTCGGGGATCTCAACCGATAGTATTTTGGCATGGCTTGACCCGTGTCCTAATGGGTCAGTTTTGTAT
GTGTGTTTTGGAAGTCAGAAGTTGCTGAAAACGGCACAAATGGAAGCCCTGTCTACTGCGCTTGAGCAAAGCGGTATAAAATTCATTTG
GGTGGTCAAATCGCCAACGGCCCAACAAGTGGCTAACGGGTACGGATCTGTCTCGGACGTATTTGAAAAACAGGTCTCCGGTAGAGG
GTTGATTATTAAAGGATGGGCCCCGCAAGTGTCAATACTCAATCATCGGGCCGTAGGTGGCTTTCTTAGTCATTGCGGATGGAACTCG
GCTTTGGAAGCTATTGTATCTGGGGTGATGATATTGGGCTGGCCCATGGAAGCGGACCAATTTGTGAATGCTAAACTGTTGGTAGACTA

CATGGGTGTTGCCATTCGAGTGTGTGAGGGCCCAGATATGGTGCCCGACTCGTCTGAGTTGGCTCAAAAAGTTGCTGAGTCGGTGAGT
GCGGAGATAGCTGA

>UGT-14
CCACAATCGACTCAAGCACTGAGTTCCACCCGCAGTGAGTCAAAAAGGCGCCCACGGCTCGGTGACTCAGTATTGATACTTGTGGGGC
CCATCCTTTTATCACAAGTCCCCTCCCAGCCACACGATCTTCGAACCCCGATGGGATCACGCCGAATTGTCCCTCCACGTGTCCCTTGG
TGGGGATTTTGGAGGACCAGAGAAACTTGACGCCACTTTTTTCCAATCCCAATGCCAATTCCTCCATTTGCTTGTTGGTCAACACCGCTT
GACTTCCAAAGCAAACGTAGACTACCGTTCGATCTTCAAATTTGTCAAGCCAAGATGTGATCTCACTATCCAAAACCAAGCTGGACCCAC
CTCTCGAAACCCGGTCATTTTCCGGTGGAAGCAAAGGTCCAACCGGCCACACCCGGTCATGACCCAAAGATTCCTTCAAATAGTCCAAA
TAAACTCGTTCCAACTCACTAAACGAGTTTATCACGAGTCCCCAACTCGCTATATTACCTCGAAACAAATCTTTGAGAAATTCCGATTGTGG
GTCTCCGGCGACGTAGCTCCGATACACCGTTGACAACTGCCACCAAGGGTAAACTGGGGAATTCGGAATTTCTGGGAAAGAAATCAGCT
CATTCTCATTGCAGCGATCATTTCTTTGCCGCATATCACGCCAAAGGGCAAAAATAACGGACATAGCCAAAGCGCCGGACGGAGAAAAAA
CGAACCGGCGAATACTGAGCTGGCAGGCGAGGCGGTGGGTCCAGCCCAAAAACATGTCGGAAATTATGGCCACCGGCGGCGATGGTT
GGCTCTGAAACCAATTGAGTATAGGATTGTAGAGTTCCCCCAAGGCAGTCATCATAGCACGGAAACCACCGGCGGGGAGGTCTTTCACG
TTTTCTACGCCGTCCGGAAG

>UGT-15
ATCATTACCCTTCCGTTCCCCAAAAACGTCGATGGACTACCATCAGGCATAGAGAGCACAGACAAACTCCCCTCTATGTCCCTCTTTGT
CCCATTTGCCACCTCCACAAAACTAATGAAACCCGATTTCGAAAATGCATTAAGCACCCTCCCACACGTAACTTTCATGGTAACTGATGG
TTTCCTAGGCTGGACCCTCGATTCGGCCTCCAAATTTGGTATTCCCCGGCTTGTAACGTATGGTTTTAACAATTACGTTGGTGTGATAAG
CCGACAAGTATCAATGAGCCAGCTTCTTAAGCAAACTGAATCAGACGATGAATTATTTCAAGTCCCGAGCTTTCCATGGATTAAGCTCAC
AAGAAATGATTTTAGCCCGCCTTTCACTGACCGCGAACCTAAAGGTCCTCACTTTGAGTTCATTATGGAGCAAGTGATTGCCACATCCC
AAAGCTACGGTCTTATAGTGAATAGTTCCTATGAGCTTGAGCCTATGTTTTTGGAATACTGGAACCGCGAATCCCACCCGAAAGCATGG

TGCGTTGGGCCTCTTTGTGTGGCAGAACCACGAAAAGAAACGAGCCAAAAGCCACCGTGGGTGAAGTGGCTAGACCAGAAGCTAGAA
CAGGGGAGTAGCCCGGTTCTTTACGTGGCATTTGGGTCTCAAGCGGAGATATCAAGGGAACAGATTGAGGAAATAAAGATTGGGCTAG
ATAAATCAGGGGTGAATTTTTTTGTGGGTGGGGGGAGAAACGAGAATGCATTGGATGATGGGTATGAAGAGAGGGTGAAGGGTAGAG
GAATTGTGGTGAGAGAGTGGGTTGAGCAAAGGGAGATATTAGGTCACGAGATTGTGAAAGGGTTTTTGAGTCATTGCGGGTGGAACTC
GGTGTCGGAGAGTATATGCGCCAAGGTGCCGATACTGGCGTGGCCGATGATGGCAGACCAGCCGCTGAATGCGAGGATGGTGGTGG
AGGAGATAAAGGTGGGGCTAAGGGTGGAGACGTGTGATGGGACGGTGAATGGGTTTGTGAAGGGAGAGGGTTTGGAGAAGATGGTG
AGGGAATTAATGGAGGGAGAGACGGGGAAGGTGGTGAGGAAGAAGGTGGCAGAGGTT
