# Supplementary material for: Transcriptomic analysis of Siberian ginseng (Eleutherococcus senticosus) to discover genes involved in saponin biosynthesis
Source: BMC Genomics. 2015 Mar 14;16(1):180. doi: 10.1186/s12864-015-1357-z (PMC4369101; doi:10.1186/s12864-015-1357-z)
Supplement: Additional file 7: — List of UGTs gene-specific primer sequences used for qPCR analysis. [file 12864_2015_1357_MOESM7_ESM.pdf]

Additional file 7. List of UGTs gene-specific primer sequences used for qPCR analysis.

Genes	Primers	
EsUGT-01	F: TGTCCATCTTGCACCCGTAA	R: CAGAGCAACCAAAGCGGTTC
EsUGT-02	F: AGAAGGTGGAGAGAGGGCAT	R: ATACACGCACTCCCAAAGCA
EsUGT-03	F: CTAAACTACTCGCCCAGCGT	R: CAGAGCAACCAAAGCGGTTC
EsUGT-04	F: TATATGGAGGTTTGGCGAGCA	R: TTCTCCCCCTCCATAACTTCCT
EsUGT-05	F: AGAGGCCCTAGATTCCGATACA	R: ATGGAATGAGCGGCCCTATG
EsUGT-06	F: AAAAGAGTTCGGTCGAGGCA	R: TTGGCTCAGACATAACCGGC
EsUGT-07	F: ACTTTGCGAGTCCGTCAGAA	R: CGAGACTTTCAGCAGCATCG
EsUGT-08	F: AATGGTGGCATTTGCAGCTC	R: TAGGCCGTCGGAGTAGAACT
EsUGT-09	F: ATTAGCCCTTGTGCCCTCTG	R: AATGCTAGGCAGATCACGGC
EsUGT-10	F: GCCATTGGTTTGGATGGATCG	R: GGCAAGCAAGCAAGCAAAAGA
EsUGT-11	F: TGCTACTAGTCCAGACGGGG	R: TCGATCTTGACAAACCCTCG
EsUGT-12	F: TTGATAACGGTAGGGGTTCCA	R: AGCACATGAGCCCATCATCG
EsUGT-13	F: AATAGTGCGAGTTGGGGGTG	R: ACACCCAACAGACTAAGCGG
EsUGT-14	F: GCTTTGGAAGTCAAGCGGTG	R: AATTGTCCCTCCACGTGTCC
EsUGT-15	F: AGCACAGACAAACTCCCCTC	R: CATACGTTACAAGCCGGGGA
EsBAS	F: GTAGCCCGGGAGAGCTAGA	R: GTAGTGGCGGCCTCAAAAGT
Actin	F: CTCGCATCTCTCAGCACCTT	R: CCACAGCCAACTGAGTTCACA
